# Polygenic score for C-reactive protein is linked to faster cortical thinning and psychopathology risk in adolescents

**DOI:** 10.1038/s44220-026-00585-w

**Published:** 2026-02-16

**Authors:** Haixia Zheng, Jonathan Savitz, Ebrahim Haroon, Jonathan Ahern, Robert J. Loughnan, Firas Naber, Bohan Xu, Katherine L. Forthman, Robin L. Aupperle, Leanne M. Williams, Martin P. Paulus, Chun Chieh Fan, Wesley K. Thompson

**Affiliations:** 1 Laureate Institute for Brain Research, Tulsa, OK USA; 2 Oxley College of Health and Natural Sciences, The University of Tulsa, Tulsa, OK USA; 3 Department of Psychiatry and Behavioral Sciences, Emory University School of Medicine, Atlanta, GA USA; 4 Center for Population Neuroscience and Genetics, Tulsa, OK USA; 5 Department of Cognitive Science, University of California, San Diego, La Jolla, CA USA; 6 J. Craig Venter Institute, La Jolla, CA USA; 7 Psychiatry and Behavioral Sciences, Stanford University School of Medicine, Palo Alto, CA USA; 8 Department of Radiology, University of California School of Medicine, San Diego, CA USA

**Keywords:** Risk factors, Developmental neurogenesis

## Abstract

Adolescence is a sensitive period of brain development marked by rapid cortical thinning and increased risk for psychiatric disorders, yet the biological drivers of atypical trajectories remain unclear. Here, using longitudinal data from the Adolescent Brain Cognitive Development Study, we examined whether genetic predisposition to systemic inflammation, indexed by polygenic scores for C-reactive protein (PGS-CRP), influences brain development and psychopathology. Higher PGS-CRP was associated with accelerated cortical thinning, particularly in medial temporal and insular regions, and with increased externalizing symptoms. Early-life infections independently predicted greater depressive and externalizing symptoms but did not interact with genetic risk. Mediation analyses indicated that cortical thinning partially accounted for the association between PGS-CRP and externalizing psychopathology. Biological annotation further identified the regional similarity between cortical effects of PGS-CRP and several neurotransmitter systems. Together, these findings suggest that genetic susceptibility to inflammation may shape adolescent brain maturation and contribute to mental health vulnerability via neuroimmune pathways.

## Abstract (web summary)

This study investigates whether genetic predisposition to systemic inflammation affects cortical thinning trajectories and the risk for psychopathology in adolescents, using data from the Adolescent Brain Cognitive Development Study.

## Main

Adolescence is an important neurodevelopmental period during which rapid cortical structure change occurs^1–3^ and when many psychiatric disorders begin to emerge^4^, suggesting that deviation from typical neuroanatomical development may partially underpin worse mental health outcomes in this sensitive age range. Among magnetic resonance imaging (MRI)-derived brain structural measures, cortical thickness exhibits a well-characterized, monotonic reduction across adolescence^1,2^, reflecting key neurodevelopmental processes such as synaptic pruning, myelination, neurogenesis and synaptogenesis^3,5–8^. By contrast, cortical surface area and gray-matter volume exhibit more dynamic and regionally variable trajectories, showing nonlinear patterns with periods of expansion, plateau or decline depending on age, sex and cortical region^3,9,10^. Recent large-scale longitudinal imaging studies suggest that cortical thinning is the dominant and most developmentally sensitive MRI marker during adolescence^3,9,10^. Aberrant patterns of cortical thickness change over time, especially accelerated thinning in frontal regions, are associated with worse cognitive and psychopathological outcomes in adolescents^11–15^. However, the upstream biological factors that drive the deviation from typical cortical thinning process remain poorly understood. Identifying these factors and their interactions is critical for understanding the origins of mental health disorders that often arise during the sensitive developmental period of adolescence.

Emerging evidence suggests that immune signaling may play a key role in neurodevelopment. The neurodevelopmental processes underlying cortical thickness change (that is, synaptic pruning, myelination, neurogenesis and synaptogenesis^3,5–8^) are highly susceptible to immune activation, which can result in lasting alterations in neural circuitry and brain function^7,16,17^. At a cellular level, immune activation influences cortical development via glial cells, such as astrocytes and microglia. These glial cells not only support neuronal health but also actively participate in synaptic pruning and neural circuit formation^18,19^. Inflammatory signals originating in the periphery can reach the brain via multiple pathways, including cytokine transport across the blood–brain barrier, vagal nerve signaling and monocyte trafficking^20^, altering microglia activation^21^. These interactions also potentially disrupt neurotransmitter systems (for example, serotonin, dopamine and glutamate) and perturb neurocircuits involved in mood regulation^20^. Indeed, neuroimaging studies have consistently shown that inflammation is associated with disrupted brain circuits integral to motivation, emotion regulation and cognitive processing^22^. Consistent with these mechanistic links, genetic and epigenetic studies have suggested that inflammation-related genetic profiles may influence risk for externalizing and internalizing psychopathology during childhood and adolescence by shaping neurocognitive development^12,23,24^.

C-reactive protein (CRP), an acute-phase protein synthesized by the liver, is one of the most widely studied peripheral markers of systemic inflammation. CRP levels are a downstream acute-phase reactant that integrates multiple upstream cytokine pathways (for example, interleukin-6, interleukin-1β and tumor necrosis factor)^25–27^ and exhibits strong correspondence between peripheral and central concentrations (*r* = 0.855)^28^, potentially explaining why peripheral CRP concentration has been associated with structural brain alterations^29,30^ and various psychiatric disorders, including major depressive disorder^31,32^, bipolar disorder^33^ and schizophrenia^34^. While circulating CRP levels reflect active inflammation at a specific time point, they are also influenced by non-specific factors, such as socioeconomic disparities, age, body mass index, smoking and sleep disturbance, making direct comparisons of inflammation challenging^35^. By contrast, the polygenic score for CRP (PGS-CRP)—a weighted sum based on an individual’s genotype that quantifies genetic predisposition for elevated CRP levels—provides a static estimate capturing an individual’s risk for inflammation. By capturing the heritable component of CRP levels, PRS-CRP (explaining approximately 16% of the variance in plasma CRP levels) is less confounded by short-term environmental or behavioral influences^36^. Moreover, CRP is the only inflammatory biomarker with sufficiently powered genome-wide association data (>500,000 participants) to support reliable polygenic scoring, making it the most robust and generalizable genetic proxy for systemic inflammatory activity^36^. Recent work shows that higher PGS-CRP is associated with greater negative affect and anxiety symptoms in adult population cohorts (*N* = 55,098), underscoring its relevance for mood disorder risk^37^. Thus, PGS-CRP provides a powerful genetic instrument to investigate how inherited pro-inflammatory tendencies influence brain development and psychiatric vulnerability.

In parallel, early-life infection provides an environmental index of immune challenge that may amplify vulnerability to psychopathology. Large epidemiological studies have found that childhood infections are associated with elevated risk of mood and psychotic disorders in adolescence and adulthood, with hospital-treated infections linked to up to a 62% higher risk of mood disorders later in life^38,39^. Importantly, the first year of life represents a critical window of neuroimmune development, when the immune system and brain undergo rapid co-maturation^40^. During this period, systemic infection may disrupt neural cell genesis and microglial programming, producing long-term alterations in brain development^41^. Supporting this, longitudinal cohorts have reported that infants exposed to antibiotic drugs in the first 2 years of life have been associated with greater risk for psychiatric diagnoses in later childhood^42^. For these reasons, our study focuses specifically on infection in the first year of life as an environmental predictor, complementing genetic liability for inflammation.

Taken together, both inherited and early environmental influences on inflammation may converge on neurodevelopmental pathways that confer risk for adolescent psychopathology. We therefore examine whether genetic liability to systemic inflammation (PGS-CRP) and early-life infection—individually and interactively—contribute to patterns of cortical thinning and emerging psychiatric symptoms in adolescence. We hypothesized that (1) greater genetic predisposition for elevated CRP levels is associated with an increased psychopathology risk and with altered patterns of cortical thinning, (2) genetic risk interacts with early-life infection to influence the psychopathology outcomes and cortical thinning process, and (3) neurobiological processes associated with cortical thinning partially mediate the relationship between genetic predisposition for high CRP levels and psychopathology. Finally, we explored whether the spatial pattern of cortical thinning overlaps with existing positron emission tomography (PET)-derived neurotransmitter receptor gradient maps, providing a biological annotation of the observed effects.

## Results

**Fig. 1:**
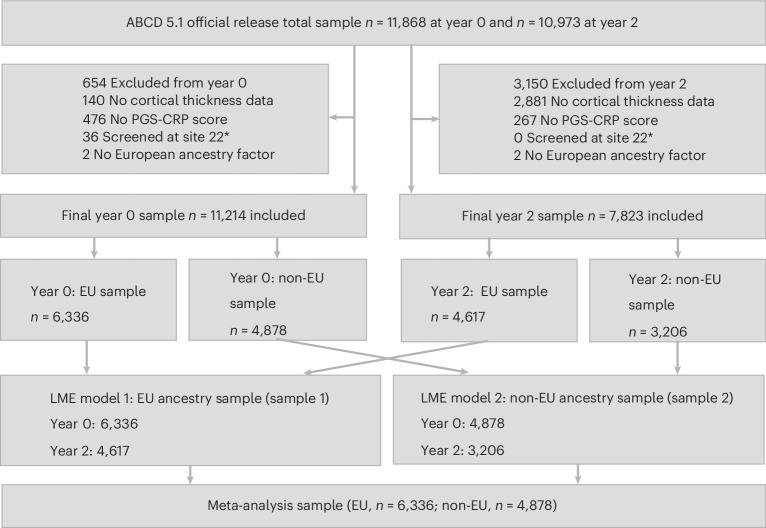
Participant flow and analytic sample derivation. Flowchart illustrating inclusion and exclusion of participants from the ABCD Study release 5.1 (total *n* = 11,868). Following quality control for cortical thickness and polygenic score (PGS-CRP) data, 6,336 European ancestry (EU) and 4,878 non-European ancestry (non-EU) participants remained at year 0. At year 2, follow-up data were available for 4,617 EU and 3,206 non-EU participants. Ancestry groups were analyzed separately using LME models and combined via meta-analysis. ^*^Site 22 was excluded because it withdrew from the ABCD Study.

Participants were stratified by imputed genetic ancestry into European and non-European samples to account for population stratification, and results were integrated using meta-analysis to enhance generalizability across diverse populations (Fig. 1). Statistical significance was determined using a false discovery rate (FDR) threshold of *P* < 0.05 based on meta-analysis results. See Methods for more details.

### PGS-CRP effects on cortical thinning and psychopathology

**Fig. 2:**
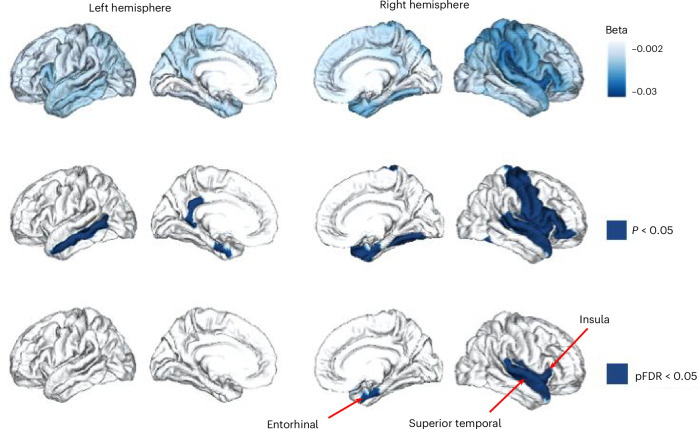
PGS-CRP is associated with variations in age-related cortical thinning during adolescence, with the strongest acceleration effect in the medial temporal lobe. This figure illustrates the interaction between PGS-CRP and age. The top row shows the standardized regression coefficients (*β*_3_) for the interaction term (age × PGS-CRP), with darker blue indicating stronger associations with accelerated cortical thinning. The middle row highlights regions where the interaction term was significant at meta-analysis *P* < 0.05), and the bottom row identifies regions that survive FDR correction in the meta-analysis (*P*_FDR_ < 0.05). Statistical analysis used two-sided LME models with individual, family and site as random intercepts. Effects reflect standardized regression coefficients (beta/*β*) from ancestry-stratified analyses, combined using inverse-weighted meta-analysis. Multiple comparisons across regions were controlled using Benjamini–Hochberg FDR. Exact *P* values and 95% confidence intervals are provided in Supplementary Table 1.

The interaction term (*β*_3_; see Methods ‘Statistical analyses’ for more details) allowed us to determine whether genetic predisposition for systemic inflammation, as captured by PGS-CRP, modifies age-related cortical thinning slopes. Meta-analytic findings across imputed ancestry groups revealed that higher PGS-CRP was significantly associated with steeper cortical thinning trajectories over 2 years across the brain cortex and particularly in medial temporal regions (Fig. 2). As shown in Fig. 2, three regions passed our statistical threshold (meta-analysis *P*_FDR_ < 0.05), including the right entorhinal cortex (PGS-CRP × age interaction effect, *β*_3_ = −0.016, SE = 0.005, *P*_FDR_ < 0.05), right insula cortex (PGS-CRP × age interaction effect, *β*_3_ = −0.018, SE = 0.005, *P*_FDR_ < 0.05) and right superior temporal gyrus (PGS-CRP × age interaction effect, *β*_3_ = −0.013, SE = 0.004, *P*_FDR_ < 0.05). These findings suggest that individuals in Adolescent Brain Cognitive Development (ABCD) with a higher genetic risk for systemic inflammation experience steeper cortical thinning trajectories in these regions over 2 years period. Regions that survived FDR correction are presented in Table 1, while the full set of results is available in Supplementary Table 1.

**Table 1:** Brain regions with significant PGS-CRP by age interaction effects on cortical thinning

Regions	Data source^1^	*β*^2^	SE^3^	*P*	*P*_FDR_
**Right entorhinal cortex**^**4**^	**Meta analysis**	**−0.016**	**0.005**	**0.002**	**0.049**
Right entorhinal cortex	EU sample	−0.019	0.007	0.008	0.493
Right entorhinal cortex	Non-EU sample	−0.014	0.008	0.098	0.308
**Right insula cortex**	**Meta analysis**	**−0.018**	**0.005**	**0.001**	**0.049**
Right insula cortex	EU sample	−0.016	0.007	0.028	0.493
Right insula cortex	Non-EU sample	−0.021	0.008	0.010	0.104
**Right superior temporal gyrus**	**Meta analysis**	**−0.013**	**0.004**	**0.002**	**0.049**
Right superior temporal gyrus	EU sample	−0.012	0.005	0.024	0.493
Right superior temporal gyrus	Non-EU sample	−0.015	0.007	0.026	0.139

^1^Participants were stratified by genetic ancestry into two groups to address population stratification: individuals of European ancestry (EU sample) and non-European ancestry (non-EU sample). LME models were performed separately in each group, and a meta-analysis was conducted to integrate findings across both groups. Statistical significance was determined through meta-analysis, with a threshold of *P* < 0.05 after FDR correction.

^2^*β*, beta coefficient of PGS-CRP by age interaction effect on cortical thickness.

^3^SE, standard error.

^4^Bolded rows indicate statistically significant results (*P*_FDR_ < 0.05).

We observed a significant association between the PGS-CRP scores and externalizing psychopathology. Specifically, higher PGS-CRP scores were associated with greater externalizing symptoms at baseline, including behavioral problems such as aggression or rule-breaking (main effect of PGS-CRP on externalizing, *β*_2_ = 0.167, SE = 0.069, *P*_FDR_ = 0.048). This suggests that genetic liability for systemic inflammation is associated with higher baseline levels of externalizing psychopathology, independent of age and early-life infection status. There were no significant main PGS-CRP effects on depression or internalizing psychopathology. The full set of results is available in Supplementary Table 2.

### Early-life infection effects on cortical thinning and psychopathology

Meta-analysis showed no significant effects of early-life infection on cortical thickness at baseline (*β*_4_), nor any interaction with age (*β*_5_), PGS-CRP (*β*_6_) or the 3-way interaction with age (*β*_7_), indicating that early-life infection did not alter cortical thinning trajectories. By contrast, participants who had reported history of early-life infection exhibited significantly higher baseline depression scores (main effect of early-life infection, *β*_4_ = 0.511, SE = 0.232, *P*_FDR_ = 0.042) and higher baseline externalizing psychopathology (*β*_4_ = 0.589, SE = 0.232, *P*_FDR_ = 0.034). The association between early-life infection and internalizing psychopathology at baseline was not significant (*β*_4_ = 0.608, SE = 0.363, *P*_FDR_ = 0.094). No interactions involving early-life infection ((*β*_5_–*β*_7_) were significant for any psychopathology outcomes. Detailed results are available in Supplementary Table 3.

### Mediation pathways

**Fig. 3:**
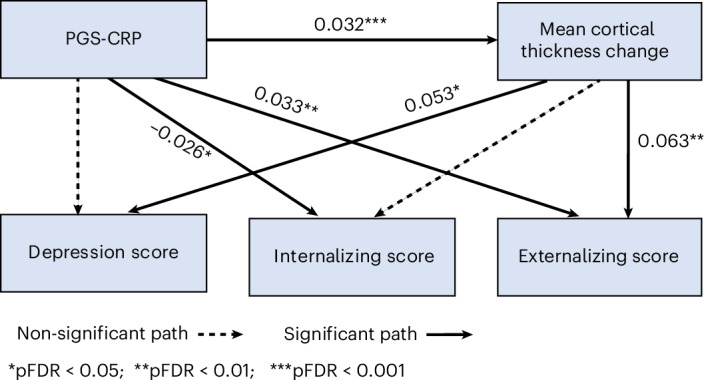
Path diagram illustrating the association between PGS-CRP and externalizing psychopathology, partially mediated by mean cortical thickness change. The pathway demonstrates that higher PGS-CRP was associated with accelerated cortical thinning, which, in turn, predicted increased externalizing symptoms. However, the mediation was partial (4% of total effect), as PGS-CRP also has a direct effect on externalizing psychopathology, indicating that the behavioral outcomes are not solely explained by cortical thinning. Statistical analysis was conducted using two-sided structural equation modeling. Indirect (mediation) effects were estimated using 1,000 bootstrap samples with bias-corrected 95% confidence intervals. All reported paths include standardized coefficients (beta/*β*) with exact *P* values, including FDR-corrected *P* values (*P*_FDR_), and bootstrap confidence intervals are provided in Supplementary Table 4.

The structural equation modeling was used to determine mediation pathways, and it demonstrated excellent fit: comparative fit index = 1.00, Tucker–Lewis index = 1.00 and standardized root mean square residual = 0.000, indicating no significant misfit. As shown in Fig. 3, changes in global mean cortical thickness were associated with depressive symptoms (*β* = 0.053, *P*_FDR_ = 0.010) and externalizing symptoms at T_2_ (*β* = 0.063, *P*_FDR_ = 0.002), but not with internalizing symptoms (*β* = 0.040, *P*_FDR_ = 0.051). PGS-CRP was negatively associated with internalizing symptoms at T_2_ (*β* = −0.026, *P*_FDR_ = 0.043) but positively associated with externalizing symptoms (*β* = 0.033, *P*_FDR_ = 0.008). No significant associations were found between PGS-CRP and depressive symptoms at T_2_ (*β* = −0.0005, *P*_FDR_ = 0.966).

The indirect (mediation) effect of PGS-CRP on externalizing symptoms (*β* = 0.002, *P*_FDR_ = 0.014) and depression (*β* = 0.002, *P*_FDR_ = 0.035) through cortical thickness was significant, while the indirect effect on internalizing symptoms was not (*β* = 0.001, *P*_FDR_ = 0.072). Total effect estimates were significant between PGS-CRP and externalizing symptoms (*β* = 0.035, *P*_FDR_ = 0.005) but not internalizing symptoms (*β* = −0.024, *P*_FDR_ = 0.051) and depressive symptoms (*β* = 0.001, *P*_FDR_ = 0.966). Approximately 4% of the total effect of PGS-CRP on externalizing psychopathology was mediated through the change in cortical thickness. The detailed results are available in Supplementary Table 4.

### Biological annotation

**Fig. 4:**
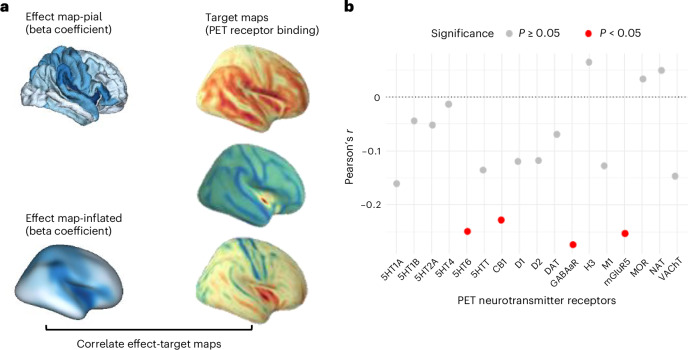
Regional association between PGS-CRP effects on cortical thinning and neurotransmitter receptor gradients. **a**, The left panel shows the regional age × PGS-CRP interaction effects (standardized *β* coefficients) mapped onto the cortical surface. The right panel shows neurotransmitter receptor distributions based on PET receptor binding for corresponding regions. These neurotransmitter maps illustrate spatial receptor gradients; the colors are not indicative of specific data values or quantitative comparisons. **b**, The scatterplot depicts Pearson’s correlation coefficients (*r*) between the regional PGS-CRP cortical-thinning effects and PET-derived neurotransmitter receptor gradients. *X*-axis labels correspond to serotonin receptors (5-HT1A, 5-HT1B, 5-HT2A, 5-HT4 and 5-HT6) and the serotonin transporter (5-HTT); the cannabinoid type-1 receptor (CB1); dopamine receptors (D1 and D2) and dopamine transporter (DAT); gamma-aminobutyric acid type-A receptor (GABAA_AA R); histamine H3 receptor (H3); muscarinic acetylcholine receptor M1 (M1) and vesicular acetylcholine transporter (VAChT); the norepinephrine transporter (NET); the metabotropic glutamate receptor 5 (mGluR5); and the mu-opioid receptor (MOR). Red points indicate significant correlations (*P*_uncorrected_ < 0.05). Statistical analysis used two-sided Pearson correlations, and spatial autocorrelation-preserving null models were used to generate significance thresholds. Exact correlation coefficients, *P* values and FDR-corrected values are provided in Supplementary Table 5.

We tested whether the regional effects of PGS-CRP on cortical thinning exhibited any correspondence with neurotransmitter receptor gradients. To explore this, we performed correlations between the effect map of PGS-CRP on cortical thinning and maps of various neurotransmitter receptor distributions. As shown in Fig. 4, our analysis revealed significant correlations between PGS-CRP effects and four specific neurotransmitter receptor gradients: serotonin (5HT_6_, *r* = −0.250, *P*_uncorrected_ = 0.010, *P*_FDR_ = 0.085), gamma-aminobutyric acid type a receptor (GABAaR, *r* = −0.274, *P*_uncorrected_ = 0.010, *P*_FDR_ = 0.085), cannabinoid (CB_1_, *r* = −0.228, *P*_uncorrected_ = 0.020, *P*_FDR_ = 0.085) and metabotropic glutamate receptor 5 (mGluR5, *r* = −0.253, *P*_uncorrected_ = 0.020, *P*_FDR_ = 0.085). Although these associations did not survive FDR correction, it highlighted that the regional effects of PGS-CRP on cortical thinning are possibly non-randomly distributed and align with neurotransmitter receptor gradients, emphasizing the role of immune-neurobiological pathways in cortical maturation and their potential contribution to psychopathology susceptibility. The full set of results is available in Supplementary Table 5.

### Sensitivity analyses

To ensure the robustness of our findings, we conducted a sensitivity analysis by combining the European (EU) and non-European (non-EU) samples into a single full-sample analysis, rather than separating them and performing a meta-analysis. The results of the full-sample analysis were consistent with the meta-analytic approach, demonstrating similar directional effects across all primary outcomes. Notably, beyond the previously identified PGS-CRP by age interaction effect in the right entorhinal cortex, right insula cortex and right superior temporal gyrus, the combined sample revealed eight additional subregions that passed the statistical threshold of *P*_FDR_ < 0.05, reflecting the increased statistical power and precision afforded by the larger sample size (detailed results are provided in Supplementary Table 6). Furthermore, the association between PGS-CRP and externalizing psychopathology at baseline remains statistically significant (detailed results are provided in Supplementary Table 7). All associations between early-life infection and psychopathology remained statistically significant (detailed results are provided in Supplementary Table 8). Consistent with our primary findings, results remained unchanged when early-life infection was redefined using a stricter threshold (≥3 days of illness). Early-life infection shows no significant impact on cortical thickness (Supplementary Table 9), but independently increased the risk for psychopathology measurements (depression, internalizing and externalizing score) at the baseline (Supplementary Table 10). When youth-reported psychopathology (Brief Problem Monitor, BPM-Y at 6 months and 2 years; Supplementary Table 11) was examined, the association between early-life infection and externalizing symptoms replicated (FDR-corrected), consistent with caregiver reports. The association between PGS-CRP and externalizing psychopathology was in the same direction but did not reach significance, a pattern consistent with known phenomenon low to modest agreement between caregiver and youth informants^43^ (Supplementary Table 11). To test whether our finding is sensitive to the selection of covariates, we re-estimated the models using the reduced model that included only ancestry-related covariates (genetic principal components), these effects remained, and eight additional subregions surpassed the FDR threshold (*P*_FDR_ < 0.05): right insula, right temporal pole, right entorhinal cortex, left entorhinal cortex, right pars opercularis, right superior temporal gyrus, left superior temporal gyrus and left pars opercularis (Supplementary Table 12). Similar to the original model, this reduced model also reproduced the association between PGS-CRP and externalizing psychopathology (*β* = 0.206, *P*_FDR_ = 0.044), while effects for internalizing and depression remained non-significant (Supplementary Table 13). These sensitivity analyses all yield consistent or stronger effects than the primary analyses, highlighting that the observed associations are robust and not dependent on the specific analytical strategy used.

## Discussion

The primary goal of this study was to examine how genetic predisposition for systemic inflammation, as measured using a PGS-CRP, influences adolescent neurodevelopment and the emergence of psychopathology, and explore its neurobiological mechanisms. Consistent with our initial hypotheses, higher genetic susceptibility to inflammation was associated with accelerated cortical thinning, particularly in medial temporal and insular regions, as well as increased externalizing psychopathology symptoms. Notably, we identified a significant indirect pathway wherein cortical thinning partially mediated the relationship between genetic predisposition to inflammation and both externalizing behaviors and depressive symptoms. Biological annotation analyses further suggested that the regions affected by PGS-CRP-related cortical thinning overlapped with neurotransmitter receptor systems enriched in serotonin, GABA, cannabinoid and glutamate signaling, highlighting the potential role of inflammation in disrupting these neurobiological pathways. These results underscore the importance of systemic inflammation as a key factor influencing neurodevelopmental trajectories during adolescence. Although the observed associations were modest in magnitude, this is consistent with the distributed and polygenic architecture of adolescent brain development and psychopathology. Small but replicable effects are common in large-scale imaging-genetic studies and are nonetheless informative for identifying biological pathways that may cumulatively shape developmental risk^10,44,45^. Thus, even subtle inflammation-related differences in cortical maturation may have meaningful population-level implications for understanding the emergence of psychiatric vulnerability.

Accelerated cortical thinning during adolescence has been linked to a range of psychiatric outcomes. In schizophrenia, for example, progressive cortical thinning, particularly in the frontotemporal cortex, is associated with symptom severity and illness duration, from the first episode of psychosis through to the chronic stages of the disorder^46,47^. Similarly, individuals with a high genetic risk for bipolar disorder show accelerated thinning and volume reduction in frontal regions, even before the onset of the disorder^48^. Extending these findings, our study suggests that genetic predisposition for inflammation, as measured by the PGS-CRP, may be a critical influencer of cortical developmental trajectories. This aligns with previous evidence linking cortical thinning during maturation to gene expression related to dendrites, dendritic spines and myelin, suggesting that shared molecular pathways may underlie both cortical development and psychiatric vulnerability^7,49,50^. In our study, the brain regions most affected by accelerated thinning—such as the entorhinal cortex, superior temporal gyrus and insula—play key roles in integrating emotional and cognitive information. The superior temporal gyrus is crucial for auditory and language comprehension^51^, the insula detects relevant stimuli and coordinates neural resources for emotional and cognitive processing^52^, and the entorhinal cortex is essential for encoding and retrieving emotional memories^53^. Moreover, the relationship between inflammation and cortical thinning appears to converge on molecular pathways involving complement component proteins such as C4A. These proteins, known for their role in synaptic pruning, have been linked to similar brain structural alterations (that is, insula and entorhinal cortex) and neurocognitive outcomes in both adolescent and adult samples^54,55^. These findings are also consistent with neuroimaging research showing that inflammation disrupts neural circuits involved in emotional regulation and cognition^22^, thus supporting a potential neurobiological mechanism by which inflammation-related genetic risk factors influence psychiatric outcomes^12,23,24^.

Building on these findings, the association between the PGS-CRP and externalizing psychopathology is notable. Externalizing behaviors, including aggression and rule-breaking, are often linked to difficulties in emotion regulation and impulse control. Our results suggest that systemic inflammation, driven by genetic factors, may impair the maturation of brain regions necessary for regulating behavior, increasing the risk for externalizing psychopathology. Importantly, these findings do not contradict the existing literature. While previous studies have primarily linked elevated inflammatory markers with fatigue, depression and stress-related disorders^20,29,56,57^—commonly associated with energy-conserving ‘sickness behavior’—our results expand this understanding by implicating systemic inflammation in difficulties with decision-making and impulse regulation, which heighten the risk for externalizing psychopathology. Furthermore, we observed a significant increase in the association between the PGS-CRP and depression scores in the non-European ancestry sample (Supplementary Table 2), consistent with previous research. This discrepancy is likely attributable to our stringent statistical approach, which prioritizes robust and conservative estimates. In addition, it is important to note that the ABCD cohort primarily consists of adolescents without diagnosed depressive disorders, which may further attenuate associations between the PGS-CRP and depression scores.

Contrary to our hypotheses, early-life infection did not interact significantly with genetic risk to affect cortical thinning trajectories. However, early-life infection independently predicted increased risk for depressive and externalizing symptoms, reinforcing previous evidence that early immunological challenges can have lasting impacts on mood disorders^38^. Our data support the ‘dual-hit’ hypothesis^58^, where early environmental stressors, such as infections, interact with genetic predispositions to heighten the risk for psychopathology later in life. The impact of early immune activation on neurodevelopment may sensitize the brain immune cell microglia (microglia priming)^59^ to subsequent inflammatory stimuli, further disrupting normal synaptic pruning or other maturation processes. The absence of a significant interaction with genetic predisposition could reflect limitations in statistical power or distinct biological pathways through which early-life infections and genetic risk independently contribute to psychopathology. In addition, our reliance on parental reporting of early-life infections introduces the possibility of recall bias and lacks detailed information regarding infection source, severity and duration. Future studies should incorporate more objective and detailed measures of early-life infection—including clinical diagnoses, infection severity, duration and specific pathogens—to improve our understanding of how these factors interact with genetic inflammation susceptibility to influence neurodevelopmental trajectories.

The biological annotations provide deeper insights into the neurobiological mechanisms at play. Specifically, we found a significant overlap between regions of cortical thinning associated with the PGS-CRP and neurotransmitter receptor gradients for serotonin, GABA, cannabinoid and glutamate signaling. This aligns with decades of animal research showing that inflammation critically modifies neurotransmitter systems, thereby increasing the risk of psychiatric disorders^58,60^. Notably, while inflammation and disruptions in serotonin and glutamate signaling have been extensively studied in relation to ‘sickness behaviors’ such as fatigue and depression^61–64^, our findings extend this perspective. Our findings, while preliminary, indicate potential neurochemical pathways through which genetic predisposition for inflammation may alter cortical development, contributing to externalizing psychopathology, which includes deficits in emotion regulation, poor impulse control and impaired decision-making.

Our findings have important clinical implications, highlighting genetic predispositions to systemic inflammation as key factors in adolescent brain development and psychopathology, opening avenues for early intervention. Targeting inflammation in at-risk individuals could help prevent psychopathology through anti-inflammatory treatments or lifestyle interventions such as exercise and dietary changes. Early identification of children with higher genetic liability for systemic inflammation may also help clinicians identify those at higher risk for neurodevelopmental disorders or psychiatric symptoms. However, several limitations of this study must be acknowledged. While we used a polygenic score for CRP to assess genetic predisposition for inflammation, it explains only a fraction of the variance in plasma CRP levels, limiting its comprehensiveness. In addition, the CRP genome-wide association study (GWAS) used to derive the polygenic score was based on data from the UK Biobank, a predominantly European adult cohort. While gene expression for phenotypes may change over time^65^, research has shown that the genetic architecture of brain phenotypes is generalizable across age^66^ and polygenic predictions for psychopathology can be effective even when the training data comes from an adult population (such as UK Biobank)^67^. It is true that there are known difficulties in translating findings from one ancestry to another^68^. We did choose polygenic scoring methods that attempt to overcome these difficulties^69^, and the convergent findings across ancestry grouping give us confidence in our results. Future research should work to include diverse populations at all levels of genetics research and should work to further validate these findings across multiple ancestries. Our findings were robust when early-life infection was redefined using a stricter threshold (≥3 days of illness), although we acknowledge that reliance on parental recall remains a limitation. Moreover, the lack of longitudinal measures of circulating CRP may have restricted our ability to capture the dynamic relationship between systemic inflammation and neurodevelopment. Future research should explore the interplay between genetic risk for inflammation and environmental pro-inflammatory factors—such as early-life adversity, viral infections or diet—to better understand their contributions to neurodevelopmental trajectories.

## Conclusion

Our findings revealed genetic predisposition to systemic inflammation, as quantified by a polygenic score for CRP, as a critical factor influencing adolescent neurodevelopmental trajectories and psychopathology risk. Specifically, elevated genetic susceptibility to inflammation was robustly associated with accelerated cortical thinning, particularly in regions vital for emotional and cognitive processing, and increased externalizing behavioral symptoms. Importantly, we identified cortical thinning as a partial mediator of the link between inflammation-related genetic risk and psychopathology, highlighting a tangible neurodevelopmental pathway through which genetic predispositions can manifest in behavioral dysfunction. While early-life infections independently heightened psychopathological risks, their interaction with genetic predisposition requires further nuanced investigation with detailed clinical data. In addition, exploratory biological annotation suggested that disruptions of neurotransmitter receptor systems (serotonin, GABA, cannabinoid and glutamate) may underpin these inflammation-linked cortical changes. Collectively, these findings highlight systemic inflammation as a potential target for early identification and preventive interventions in youth at elevated risk, offering critical insights into the complex neuroimmune mechanisms that contribute to mental health disorders emerging during adolescence. Future studies should build upon these results by integrating detailed longitudinal measures of inflammation, environmental exposures and precise mechanistic investigations to deepen our understanding of these critical developmental pathways.

## Methods

### Ethics statement

The ABCD Study obtained institutional review board approval at each participating site, and written informed consent from parents/guardians and assent from children were collected by ABCD investigators. The present study involved secondary analysis of fully de-identified data available through the NIMH Data Archive and was therefore determined not to involve human subjects research and did not require additional ethical review.

### Study design and population

This longitudinal cohort study utilized data from the ABCD Study, an ongoing large-scale sample of 11,868 youth aged 9–10 years at baseline; 47.1% were assigned female at birth. Families received monetary compensation for participation, consistent with ABCD Study protocols. Recruitment was conducted primarily through public and private elementary schools using a probability sampling approach stratified by age, sex, race/ethnicity, socioeconomic status and urbanicity, with targeted oversampling of racial/ethnic groups historically underrepresented in research (for example, Black, Hispanic and rural youth) to better reflect the US population^70^. The participating schools were drawn from each site’s catchment area, collectively encompassing ~20% of the US population of 9–10-year-olds. Approximately 9.6% of the contacted families enrolled, yielding a cohort broadly representative of the sociodemographic composition of US children^70^. Enrollment demographics were monitored throughout recruitment, and later school samples were dynamically adjusted to correct deviations from target demographics^70^. Approximately half of the sample was enriched for children with early indicators of externalizing or internalizing symptoms to ensure sufficient power for studying developmental risk trajectories^71^. At baseline (T_0_, *n* = 11,214), 99% of the participants completed the T1-weighted structural MRI. At the 2-year follow-up (T_2_, *n* = 7,823), approximately 70% of the baseline cohort have finished the second MRI scan and available in ABCD data release 5.1. Basic demographic characteristics, exposure and outcome variables for baseline, year 2 and ancestry-stratified samples are shown in Table 2. Owing to page limitations, additional socioeconomic characteristics (race/ethnicity, parental education and household income) for the full sample and genetic ancestry-stratified samples are presented in Supplementary Table 14. Demographic, clinical and structural MRI data were obtained from the National Institutes of Mental Health Data Archive (NDA). Full recruitment details and neuroimaging acquisition are available in previous publications^70,71^.

**Table 2:** Basic demographic^1^, exposure and outcome variables in full analytical sample, stratified by ancestry and year

EU sample (sample 1)	Year 0 (*n* = 6,336)	Year 2 (*n* = 4,617)
Mean cortical thickness (mean (s.d.)^2^)	2.74 (0.08)	2.70 (0.08)
Depression (mean (s.d.))	53.60 (5.66)	53.94 (6.02)
Internalizing (mean (s.d.))	48.70 (10.45)	48.30 (10.33)
Externalizing (mean (s.d.))	45.42 (10.01)	44.47 (9.65)
Polygenic score for CRP (mean (s.d.))	−0.18 (0.93)	−0.18 (0.93)
Age (years)	9.92 (0.63)	11.96 (0.65)
Sex at birth, female (%)	2,982 (47.1%)	2,080 (45.1%)
Body mass index (mean (s.d.))	17.92 (3.31)	19.69 (4.01)
**Non-EU sample (sample 2)**	**Year 0 (*n*** **=** **4,878)**	**Year 2 (*n*** **=** **3,206)**
Mean cortical thickness (mean (s.d.))	2.70 (0.08)	2.66 (0.08)
Depression (mean (s.d.))	53.61 (5.80)	53.55 (5.83)
Internalizing (mean (s.d.))	48.19 (10.91)	46.96 (10.76)
Externalizing (mean (s.d.))	46.26 (10.74)	44.81 (10.18)
Polygenic score for CRP (mean (s.d.))	0.25 (1.04)	0.24 (1.03)
Age (years)	9.90 (0.62)	11.94 (0.66)
Sex at birth, female (%)	2,347 (48.1%)	1,511 (47.1%)
Body mass index (mean (s.d.))	19.78 (4.46)	21.66 (4.80)
**Full meta-analysis sample**	**Year 0 (*n*** **=** **11,214)**	**Year 2 (*n*** **=** **7,823)**
Mean cortical thickness (mean (s.d.))	2.72 (0.08)	2.69 (0.08)
Depression (mean (s.d.))	53.60 (5.72)	53.78 (5.95)
Internalizing (mean (s.d.))	48.48 (10.66)	47.75 (10.53)
Externalizing (mean (s.d.))	45.77 (10.34)	44.63 (9.87)
Polygenic score for CRP (mean (s.d.))	0.00 (1.00)	−0.01 (1.00)
Early-life infection, yes (%)	1,710 (16.7%)	1,218 (16.9%)
Age (years)	9.91 (0.63)	11.95 (0.65)
Sex at birth, female (%)	5,329 (47.5%)	3,591 (45.9%)
Body mass index (mean (s.d.))	18.73 (3.96)	20.49 (4.45)

^1^Owing to page limitations, detailed socioeconomic variables (race/ethnicity, parental education and household income) are presented in Supplementary Table 14.

^2^s.d., standard deviation.

Exclusion criteria for the ABCD Study included non-fluency in English, not having a guardian fluent in English or Spanish, major medical or neurological conditions, gestational age below 28 weeks or birthweight under 1,200 grams, contraindications for MRI scanning, a history of traumatic brain injury, current diagnosis of schizophrenia, moderate to severe autism spectrum disorder, intellectual disability or alcohol/substance use disorder.

This report was based on data from the ABCD 5.1 data release. The data analyzed were collected between September 1, 2016, and February 15, 2021. Of the 11,868 participants in the original cohort, 140 had missing baseline (year 0) MRI data, 476 had missing PGS-CRP data, 36 were screened at a site that dropped out of the ABCD Study, and 2 had missing genetic ancestry data and were excluded, yielding a final analytic sample of 11,214. Participants were stratified by genetically inferred continental-ancestry clusters (European (*n* = 6,336) versus non-European (*n* = 4,878)) based on genetic ancestry score thresholds (≥0.8 versus <0.8). Within each ancestry group, follow-up data were available for the majority: 4,588 European ancestry and 3,158 non-European ancestry youth completed both baseline and year 2 assessments, while others did not complete year 2 primarily owing to attrition or incomplete visits. A flow diagram (Fig. 1) summarizes the number of participants at each stage of inclusion, exclusion and follow-up. In addition, missingness across exposures (PGS-CRP, early-life infection), outcomes (cortical thickness, psychopathology) and covariates is summarized in Supplementary Table 15. Attrition between baseline and year 2 is detailed in Supplementary Table 16. This report follows the Strengthening the Reporting of Observational Studies in Epidemiology (STROBE) reporting guidelines.

### Exposure variables

#### Polygenic score calculation

##### Genome-wide association data

The primary exposure variable for this report was the PGS-CRP, which provides a quantitative instrument of an individual’s genetic propensity for elevated systemic inflammation. We generated the PGS-CRP using summary statistics from the UK Biobank participants (*N* = 427,367, European descent) and the Cohorts for Heart and Aging Research in Genomic Epidemiology (CHARGE) Consortium (total *N* = 575,531 European descent)^36^ accessed from the GWAS Catalog^72^ under accession code GCST90029070 (https://www.ebi.ac.uk/gwas/studies/GCST90029070). Summary statistics were cleaned and aligned to genome build GRCh38 using the function cleansumstats^73^.

##### ABCD genetic data

Genetic data was collected using blood or saliva samples from participants of the ABCD Study^74^. A total of 656,247 genomic markers were measured using Smokescreen array^75^. To increase the amount of overlap in genetic variants between the ABCD sample and the CRP GWAS, we imputed the markers from our Smokescreen array using the TOPMED imputation server^76^. These imputed variants were fractional dosages and were converted to an integer number of alleles using a best guess threshold of 0.9 resulting in over 280 million imputed variants aligned to genome build GRCh38. After imputation target genetic data was restricted to only autosomal variants with a minor allele frequency of 1% (0.01) or greater leaving just under 11 million single nucleotide polymorphisms (SNPs) in the target data. Genetic continental ancestry was estimated using SNPweights^77^ and external genomic reference panels for African, East Asian, European^78^, and Indigenous North and South American^79^ ancestry populations. Individuals with inferred genetic ancestry at least 80% (0.8) consistent with the European ancestry reference panels were considered European ancestry (EU sample, *N* = 6,605 at baseline and *N* = 5,992 at follow-up), and all others were grouped into the non-European ancestry sample (non-EU sample, *N* = 4,500 at baseline and *N* = 3,750 at follow-up).

##### Polygenic score computation

Best practices for imputing polygenic scores in the ABCD are based on the results of a previous analysis that found that the Bayesian polygenic scoring method, PRScs^80^, maximized variance explained while maintaining a manageable computational load and improving or maintaining performance across ancestry compared with other Bayesian and pruning and thresholding methods^69^. Posterior effect sizes were generated using PRScs using 10,000 Markov Chain Monte Carlo iterations and 5,000 burnin interactions. All other parameters were left at their default. Linkage disequilibrium reference panels required for this analysis were based on data from 1000 Genomes Phase 3 and matched the ancestry of the summary statistics. Posterior effect sizes were applied to ABCD target data converted to polygenic scores using PLINK 2.0 (ref. ^81^).

### Early-life infection

The secondary exposure variable was early-life infection, operationalized as a binary indicator based on parental reports of infant illness. Specifically, parents were asked: ‘How many days in the first 12 months of life did your child have any severe infections?’ Owing to the lack of detailed diagnostic information (for example, infection type, severity or duration), we applied a binary coding approach: responses indicating no reported sick days were coded as 0 (no severe early-life infection), and those reporting one or more days of illness were coded as 1 (presence of severe early-life infection). Although limited in clinical detail, this measure provides a proxy for early-life infection as a biological stressor.

### Outcome variables

The primary outcomes of interest were MRI indices of cortical thickness and measures of psychopathology. MRI acquisition and processing adhered to the standardized protocols of the ABCD Study^71,82^. Imaging data were acquired on three types of 3T scanner—Siemens Prisma/Prisma Fit, GE MR750 and Philips Achieva dStream/Ingenia. T1-weighted images were corrected for gradient nonlinearity distortions using scanner-specific transformations, and cortical reconstruction and volumetric segmentation were performed using FreeSurfer v7.1.1, which includes procedures addressing head motion, distortion and intensity inhomogeneity^82^. Cortical thickness estimates were derived for 34 parcellations per hemisphere according to the Desikan–Killiany atlas^83^. All imaging data were previously collected and preprocessed by the ABCD Data Analysis, Informatics, and Resource Center (DAIRC), which performed both automated and manual quality control before data release. No new MRI data were generated for the present analyses.

Psychopathology was assessed using the depression, internalizing and externalizing symptom scales from the parent-reported Child Behavior Checklist (CBCL). The CBCL is a well-validated and widely used tool for evaluating youth mental health, known for its high reliability and internal consistency^84^. It consists of eight syndrome scales (anxious, depressed, somatic complaints, social problems, thought problems, attention problems, rule-breaking behavior and aggressive behavior) that load into two broad categories: internalizing and externalizing problems^85^. The internalizing and externalizing scores from CBCL are commonly used for identifying broad patterns of emotional and behavioral problems^85^. Depression was examined as a distinct outcome owing to its connection inflammatory markers in the literature and its central role in mental health disorders^57^. The continuous estimation of depression was derived from CBCL DSM-5 oriented affective problem scale, developed in the 2001 revision of the CBCL. Parents rated the presence of specific child behaviors over the past 6 months using a scale of 0 (‘not true’), 1 (‘somewhat or sometimes true’) or 2 (‘very true or often true’), with higher scores representing more notable psychopathology.

### Covariates

Sex at birth, body mass index, self-reported race/ethnicity, parental education, household income and the first ten principal components of genetic ancestry (to account for population stratification) were included as fixed-effect covariates in analyses. These covariates have been previously reported to be associated with systemic inflammation and mental health outcomes. Parental education and household income were reported by the guardian and are key indicators of socioeconomic status, known to be associated with neurodevelopmental trajectories and psychopathology^86,87^. The ten principal components of genetic ancestry were derived from the ABCD genetic data to account for population stratification across diverse ancestral backgrounds, thereby minimizing spurious associations unrelated to the exposure and outcomes of interest. The detailed methodology for computing these genetic principal components has been previously published^88^. Together, these covariates were included to control for confounds and to help isolate the unbiased effects of the primary exposure variables on the outcomes of interest.

In addition, individual, family and study site were included as random-effect covariates in the analyses to account for nesting of the data with respect to these variables. Individual random effects account for nesting of participants’ repeated measurement occasions (two visits). Many ABCD Study participants come from families with siblings also in the study (9,420 families); hence, family random effects account for nesting of data within families. Finally, there are 21 data collection sites in the ABCD Study, and site random intercepts account for nesting at this level.

### Statistical analyses

Participants were stratified by imputed genetic ancestry into two groups to account for population stratification: sample 1, individuals of European ancestry (EU sample, *N* = 6,605 at baseline and *N* = 5,992 at follow-up), and sample 2, individuals of non-European ancestry (non-EU sample, *N* = 4,500 at baseline and *N* = 3,750 at follow-up; see ‘ABCD genetic data’ for further details). This stratification was conducted to mitigate bias arising from population stratification, a phenomenon where differences in allele frequencies across ancestral groups reflect demographic history rather than trait-specific associations^68^ and to help account for known difference in polygenic score performance when translating across ancestries^68^. Given that genetic variants contributing to polygenic scores can vary in frequency and effect size between populations, stratification ensures that the polygenic scores reliably capture ancestry-specific genetic risk, thereby enhancing the validity and generalizability of the findings^68^.

A meta-analysis was conducted to integrate findings across participants of European and non-European ancestry samples. This approach was chosen to synthesize results from diverse genetic backgrounds, enhancing the generalizability of our findings and providing a robust assessment of the genetic influences on neurodevelopmental outcomes across different populations. Statistical significance was evaluated using an FDR threshold of *P* < 0.05 of meta-analysis results.

Linear mixed-effects (LME) models were used to examine whether genetic predisposition to systemic inflammation, as indexed by the PGS-CRP, was associated with cortical thickness trajectories and psychopathology risk during adolescence (primary hypothesis), and whether this association was moderated by early-life infection (secondary hypothesis). The model included a three-way interaction between PGS-CRP, age and early-life infection, with age modeled continuously to capture longitudinal change from baseline (T_0_) to follow-up (T_2_).

The model was specified as

$$\begin{array}{l}{\mathrm{Outcomes}}_{ijk}={\beta }_{0}+{\beta }_{1}\left({\mathrm{Age}}_{ijk}\right)+{\beta }_{2}\left(\mathrm{PGS}-{\mathrm{CRP}}_{i}\right)+{\beta }_{3}\left({\mathrm{Age}}_{ijk}\times \mathrm{PGS}-{\mathrm{CRP}}_{i}\right)\\ \,\,\,\,\,\,\,\,\,\,\,\,\,\,\,\,\,\,\,\,\,+{\beta }_{4}\left({\mathrm{EarlyLifeInfection}}_{i}\right)+{\beta }_{5}\left({\mathrm{Age}}_{ijk}\times {\mathrm{EarlyLifeInfection}}_{i}\right)\\ \,\,\,\,\,\,\,\,\,\,\,\,\,\,\,\,\,\,\,\,\,+{\beta }_{6}\left(\mathrm{PGS}-{\mathrm{CRP}}_{i}\times {\mathrm{EarlyLifeInfection}}_{i}\right)\\ \,\,\,\,\,\,\,\,\,\,\,\,\,\,\,\,\,\,\,\,\,+{\beta }_{7}\left({\mathrm{Age}}_{ijk}\times \mathrm{PGS}-{\mathrm{CRP}}_{i}\times {\mathrm{EarlyLifeInfection}}_{i}\right)\\ \,\,\,\,\,\,\,\,\,\,\,\,\,\,\,\,\,\,\,\,\,+{X}_{ijk}\beta +{u}_{i}+{u}_{j}+{w}_{k}+{\varepsilon }_{ijk}\end{array}\,\,$$

where *i*, *j* and *k* index individuals, families and study sites, respectively. *X* includes fixed-effect covariates: sex, body mass index, race, parental education, household income and the first ten principal components of genetic ancestry. The random effects include individual ID (*u*_*i*_), family ID (*v*_*j*_) and study site (*w*_*k*_). The *ε*_*ijk*_ represents the residual error. All continuous predictors were standardized before analysis.

### Mediation analyses

We implemented structural equation models (SEMs) to examine whether changes in cortical thickness mediate the association between PGS-CRP and psychopathology. SEMs included depressive, internalizing and externalizing symptoms at 2-year follow-up as outcome variables; each was regressed on changes in global mean cortical thickness (T_2_ – T_0_), PGS-CRP, age at T_2_, sex, baseline cortical thickness and baseline psychopathology symptoms. Global mean cortical thickness change was included as a mediator of the PGS-CRP to psychopathology relationship. Although primary analyses tested regional thickness associations, global cortical thinning was used in the mediation models to capture a unified index of brain maturation. This is supported by cortical thinning during adolescence as a global coordinated process reflecting normative neurodevelopment. Using global thickness change provides a developmentally meaningful and statistically efficient summary of individual differences in cortical maturation. One thousand bootstrap samples were used to obtain robust estimates and confidence intervals for direct and indirect effects. Model fit was evaluated using the comparative fit index, Tucker–Lewis index and standardized root mean square residual.

### Biological annotation

To explore the potential biological mechanisms underlying the effects of PGS-CRP on cortical thinning in adolescents, we conducted a neurobiological annotation analysis using the Neuromaps toolbox^89^. In this framework, ‘annotation’ refers to testing whether the spatial pattern of cortical effects associated with PGS-CRP aligns with known maps of neurotransmitter receptor density. We compared our cortical effect maps with standardized PET-derived receptor distribution maps spanning 17 receptors and transporters across 9 neurotransmitter systems. These include serotonin (5-HT_1A_^90^, 5-HT_1B_^90^, 5-HT_2A_^91^, 5-HT_4_^91^, 5-HT_6_^92^ and 5-HTT^91^), cannabinoid (CB_1_^93^), dopamine (D_1_^94^, D_2_^95^ and DAT^96^), gamma-aminobutyric acid type a receptor (GABA_A_^97^), histamine (H_3_^98^), norepinephrine (NET^99^), acetylcholine (M_1_^100^ and VAChT^101^), glutamate (mGluR_5_^102^) and opioid (MOR^103^). To rigorously assess spatial correspondence, we applied spatial autocorrelation-preserving permutation tests, termed spatial null model^89,104^. This approach generates null distributions that maintain the intrinsic spatial smoothness of cortical maps, allowing robust statistical evaluation of whether observed receptor–cortical thinning associations exceed chance. This procedure provides insights into whether genetic liability to inflammation influences cortical regions that overlap with specific neurotransmitter systems, thereby offering a molecular-level interpretation of observed neuroimaging effects.

### Reporting summary

Further information on research design is available in the Nature Portfolio Reporting Summary linked to this article.

## Supplementary information

Supplementary Information Supplementary Tables 1–16.

Reporting Summary

## Data Availability

This study used data from the Adolescent Brain Cognitive Development (ABCD) Study, curated release 5.1. These data are publicly available through the National Institute of Mental Health Data Archive (NDA) under the ABCD collection (https://nda.nih.gov/abcd). Access requires completion of the NDA Data Use Certification.

## Code availability

No custom algorithms were developed for this study. Analyses were performed using publicly available software, including PLINK 2.0 and PRS-CS for genetic analyses; FreeSurfer v7.1.1 for neuroimaging processing; and R (version 4.4.1) with the packages nlme (v3.1-168), metafor (v4.8-0) and lavaan (v0.6-19) for statistical modeling and mediation analyses. Neurobiological annotation was conducted using the Neuromaps toolbox. All software used in this study is freely accessible.
