# Supplementary material for: Polygenic score for C-reactive protein is linked to faster cortical thinning and psychopathology risk in adolescents
Source: Nat Ment Health. 2026 Feb 16;4(3):427–38. doi: 10.1038/s44220-026-00585-w (PMC12975513; doi:10.1038/s44220-026-00585-w)
Supplement: Supplementary file 1 — Supplementary Tables 1–16. [file 44220_2026_585_MOESM1_ESM.pdf]

nature mental health

Supplementary information

https://doi.org/10.1038/s44220-026-00585-w

Polygenic score for C-reactive protein is linked to faster cortical thinning and psychopathology risk in adolescents

In the format provided by the authors and unedited

Supplementary Information

This file contains Supplementary Tables S1-S16. No supplementary figures accompany this manuscript.

Contents

Supplementary Table S1. Summary of PGS-CRP by Age Interaction Effects on Cortical Thickness....................2

Supplementary Table S2. Summary of PGS-CRP Effects on Psychopathology....................10

Supplementary Table S3. Summary of Early-Life Infection Effects on Psychopathology....................10

Supplementary Table S4. Summary of the Results from Mediation Pathways....................11

Supplementary Table S5. Summary of Biological Annotation Results....................12

Supplementary Table S6. Summary of PGS-CRP by Age Interaction Effects on Cortical Thickness Using Full Samples....................13

Supplementary Table S7. Summary of PGS-CRP Effects on Psychopathology Using Full Sample....................15

Supplementary Table S8 . Summary of Early-Life Infection Effects on Psychopathology Using Full Sample....................15

Supplementary Table S9. Sensitivity Analysis examining Associations between Early-Life Infection (defined as ≥3 days of illness during infancy) and Cortical Thickness.....................16

Supplementary Table S10. Sensitivity Analysis examining Associations between Early-Life Infection (defined as ≥3 days of illness during infancy)and Psychopathology Outcomes.....................20

Supplementary Table S11. Sensitivity Analysis of the Effects of Early-Life Infection and PGS-CRP on Youth-Reported (BPM-Y) Psychopathology Outcomes.....................20

Supplementary Table S12. Sensitivity Analysis of the Interaction between PGS-CRP and Age on Cortical Brain Thickness, adjusting only for Genetic Principal Components.....................21

Supplementary Table S13. Sensitivity Analysis of the Effect of PGS-CRP on Psychopathology Outcomes, adjusting only for Genetic Principal Components.....................23

Supplementary Table S14. Demographic, exposure, and outcome variables in ancestry-stratified samples (EU and Non-EU) and full meta-analysis sample for in Year 0 and Year 2.....................24

Supplementary Table S15. Summary of Missing Data for Exposures, Outcomes, and Covariates in Baseline (Y0) and 2-Year Follow-up (Y2) Cohorts.....................27

Supplementary Table S16. Summary of Attrition between Baseline (Y0) and 2-Year Follow-up (Y2) Cohorts.....................28

Supplementary Table S1. Summary of PGS-CRP by Age Interaction Effects on Cortical Thickness

**Brain Regions**	**Source of Results**	**β**[1]	**SE**[2]	***p***	***p*.FDR**[3]
Left Banks of Superior Temporal Sulcus	Meta-analysis	-0.002	0.004	0.511	0.749
Left caudalanteriorcingulate	Meta-analysis	0.000	0.004	0.891	0.917
Left caudalmiddlefrontal	Meta-analysis	0.000	0.005	0.976	0.976
Left cuneus	Meta-analysis	0.000	0.004	0.892	0.917
Left entorhinal	Meta-analysis	-0.014	0.005	0.011	0.153
Left frontalpole	Meta-analysis	-0.004	0.012	0.708	0.838
Left fusiform	Meta-analysis	-0.003	0.004	0.512	0.749
Left inferiorparietal	Meta-analysis	-0.008	0.005	0.114	0.386
Left inferiortemporal	Meta-analysis	-0.006	0.005	0.165	0.489
Left insula	Meta-analysis	-0.006	0.005	0.299	0.644
Left isthmuscingulate	Meta-analysis	-0.007	0.004	0.067	0.303
Left lateraloccipital	Meta-analysis	-0.001	0.004	0.769	0.858
Left lateralorbitofrontal	Meta-analysis	-0.004	0.005	0.401	0.730
Left lingual	Meta-analysis	0.004	0.007	0.549	0.749
Left medialorbitofrontal	Meta-analysis	0.003	0.005	0.590	0.749
Left middletemporal	Meta-analysis	-0.009	0.005	0.058	0.298
Left paracentral	Meta-analysis	-0.007	0.007	0.294	0.644
Left parahippocampal	Meta-analysis	0.000	0.004	0.904	0.917
Left parsopercularis	Meta-analysis	-0.009	0.004	0.033	0.285
Left parsorbitalis	Meta-analysis	-0.002	0.004	0.576	0.749
Left parstriangularis	Meta-analysis	-0.003	0.005	0.536	0.749
Left pericalcarine	Meta-analysis	0.005	0.004	0.226	0.553
Left postcentral	Meta-analysis	-0.002	0.004	0.591	0.749
Left posteriorcingulate	Meta-analysis	-0.003	0.005	0.590	0.749
Left precentral	Meta-analysis	-0.003	0.005	0.502	0.749
Left precuneus	Meta-analysis	-0.004	0.004	0.373	0.719

[1] β = Beta coefficient of PGS-CRP by Age Interaction Effects on Cortical Thickness
[2] SE = Standard Error
[3] p.FDR = p-value adjusted for False Discovery Rate

Left rostralanteriorcingulate	Meta-analysis	0.005	0.005	0.285	0.644
Left rostralmiddlefrontal	Meta-analysis	-0.001	0.005	0.792	0.865
Left superiorfrontal	Meta-analysis	-0.004	0.004	0.386	0.722
Left superiorparietal	Meta-analysis	-0.008	0.006	0.200	0.530
Left superiortemporal	Meta-analysis	-0.007	0.004	0.083	0.311
Left supramarginal	Meta-analysis	-0.005	0.006	0.427	0.749
Left temporalpole	Meta-analysis	-0.009	0.006	0.135	0.437
Left transversetemporal	Meta-analysis	-0.003	0.004	0.375	0.719
Mean cortical thickness in mm for left hemisphere	Meta-analysis	-0.006	0.004	0.187	0.530
Mean cortical thickness in mm for right hemisphere	Meta-analysis	-0.010	0.004	0.023	0.231
Mean cortical thickness in mm for whole brain	Meta-analysis	-0.008	0.004	0.059	0.298
Right Banks of Superior Temporal Sulcus	Meta-analysis	-0.006	0.003	0.076	0.303
Right caudalanteriorcingulate	Meta-analysis	-0.002	0.006	0.702	0.838
Right caudalmiddlefrontal	Meta-analysis	-0.005	0.005	0.324	0.677
Right cuneus	Meta-analysis	0.002	0.004	0.538	0.749
Right entorhinal	Meta-analysis	-0.016	0.005	0.002	0.049
Right frontalpole	Meta-analysis	0.002	0.008	0.773	0.858
Right fusiform	Meta-analysis	-0.007	0.004	0.112	0.386
Right inferiorparietal	Meta-analysis	-0.006	0.011	0.602	0.749
Right inferiortemporal	Meta-analysis	-0.008	0.004	0.074	0.303
Right insula	Meta-analysis	-0.018	0.006	0.001	0.049
Right isthmuscingulate	Meta-analysis	-0.008	0.007	0.222	0.553
Right lateraloccipital	Meta-analysis	-0.002	0.005	0.720	0.838
Right lateralorbitofrontal	Meta-analysis	-0.001	0.005	0.817	0.870
Right lingual	Meta-analysis	0.003	0.005	0.463	0.749
Right medialorbitofrontal	Meta-analysis	-0.001	0.005	0.821	0.870
Right middletemporal	Meta-analysis	-0.003	0.004	0.534	0.749
Right paracentral	Meta-analysis	-0.003	0.004	0.446	0.749
Right parahippocampal	Meta-analysis	-0.002	0.005	0.665	0.815
Right parsopercularis	Meta-analysis	-0.008	0.004	0.051	0.298
Right parsorbitalis	Meta-analysis	-0.011	0.006	0.044	0.285
Right parstriangularis	Meta-analysis	-0.013	0.007	0.043	0.285
Right pericalcarine	Meta-analysis	0.003	0.006	0.586	0.749
Right postcentral	Meta-analysis	-0.011	0.005	0.022	0.231

Right posteriorcingulate	Meta-analysis	-0.005	0.008	0.523	0.749
Right precentral	Meta-analysis	-0.010	0.005	0.042	0.285
Right precuneus	Meta-analysis	-0.003	0.004	0.488	0.749
Right rostralanteriorcingulate	Meta-analysis	-0.003	0.009	0.745	0.853
Right rostralmiddlefrontal	Meta-analysis	-0.005	0.005	0.373	0.719
Right superiorfrontal	Meta-analysis	-0.005	0.005	0.265	0.628
Right superiorparietal	Meta-analysis	-0.012	0.007	0.077	0.303
Right superiortemporal	Meta-analysis	-0.013	0.004	0.002	0.049
Right supramarginal	Meta-analysis	-0.015	0.012	0.202	0.530
Right temporalpole	Meta-analysis	-0.017	0.006	0.005	0.082
Right transversetemporal	Meta-analysis	-0.006	0.004	0.144	0.446

**Brain Regions**	**Source of Results**	**β**	**SE**	***p***	***p*.FDR**
Left Banks of Superior Temporal Sulcus	EU Sample	0.000	0.005	0.950	0.986
Left caudalanteriorcingulate	EU Sample	0.000	0.005	0.959	0.986
Left caudalmiddlefrontal	EU Sample	-0.004	0.006	0.576	0.926
Left cuneus	EU Sample	-0.001	0.005	0.831	0.986
Left entorhinal	EU Sample	-0.015	0.007	0.036	0.516
Left frontalpole	EU Sample	0.007	0.006	0.248	0.836
Left fusiform	EU Sample	-0.004	0.006	0.440	0.926
Left inferiorparietal	EU Sample	-0.004	0.006	0.586	0.926
Left inferiortemporal	EU Sample	-0.004	0.006	0.473	0.926
Left insula	EU Sample	-0.004	0.008	0.621	0.928
Left isthmuscingulate	EU Sample	-0.004	0.004	0.380	0.926
Left lateraloccipital	EU Sample	0.000	0.005	0.950	0.986
Left lateralorbitofrontal	EU Sample	0.000	0.006	0.983	0.990
Left lingual	EU Sample	-0.003	0.005	0.587	0.926
Left medialorbitofrontal	EU Sample	0.001	0.007	0.907	0.986
Left middletemporal	EU Sample	-0.007	0.006	0.207	0.836
Left paracentral	EU Sample	-0.001	0.006	0.908	0.986
Left parahippocampal	EU Sample	-0.003	0.004	0.400	0.926
Left parsopercularis	EU Sample	-0.007	0.006	0.217	0.836
Left parsorbitalis	EU Sample	-0.005	0.005	0.374	0.926
Left parstriangularis	EU Sample	0.000	0.006	0.990	0.990
Left pericalcarine	EU Sample	0.003	0.005	0.510	0.926
Left postcentral	EU Sample	-0.002	0.005	0.655	0.928

Left posteriorcingulate	EU Sample	0.002	0.004	0.666	0.928
Left precentral	EU Sample	-0.001	0.006	0.903	0.986
Left precuneus	EU Sample	-0.001	0.005	0.886	0.986
Left rostralanteriorcingulate	EU Sample	0.007	0.007	0.307	0.926
Left rostralmiddlefrontal	EU Sample	0.003	0.006	0.609	0.928
Left superiorfrontal	EU Sample	-0.003	0.006	0.648	0.928
Left superiorparietal	EU Sample	-0.002	0.006	0.733	0.982
Left superiortemporal	EU Sample	-0.008	0.005	0.116	0.822
Left supramarginal	EU Sample	0.001	0.006	0.918	0.986
Left temporalpole	EU Sample	-0.006	0.008	0.416	0.926
Left transversetemporal	EU Sample	-0.003	0.005	0.587	0.926
Mean cortical thickness in mm for left hemisphere	EU Sample	-0.004	0.006	0.497	0.926
Mean cortical thickness in mm for right hemisphere	EU Sample	-0.006	0.006	0.257	0.836
Mean cortical thickness in mm for whole brain	EU Sample	-0.005	0.005	0.341	0.926
Right Banks of Superior Temporal Sulcus	EU Sample	-0.006	0.005	0.186	0.836
Right caudalanteriorcingulate	EU Sample	0.004	0.004	0.408	0.926
Right caudalmiddlefrontal	EU Sample	-0.009	0.007	0.202	0.836
Right cuneus	EU Sample	0.000	0.005	0.956	0.986
Right entorhinal	EU Sample	-0.019	0.007	0.008	0.493
Right frontalpole	EU Sample	0.010	0.006	0.112	0.822
Right fusiform	EU Sample	-0.009	0.006	0.114	0.822
Right inferiorparietal	EU Sample	0.005	0.006	0.430	0.926
Right inferiortemporal	EU Sample	-0.006	0.006	0.259	0.836
Right insula	EU Sample	-0.016	0.007	0.028	0.493
Right isthmuscingulate	EU Sample	-0.002	0.004	0.689	0.941
Right lateraloccipital	EU Sample	0.003	0.005	0.527	0.926
Right lateralorbitofrontal	EU Sample	0.002	0.007	0.784	0.986
Right lingual	EU Sample	-0.001	0.005	0.872	0.986
Right medialorbitofrontal	EU Sample	0.001	0.007	0.860	0.986
Right middletemporal	EU Sample	0.000	0.006	0.952	0.986
Right paracentral	EU Sample	-0.001	0.006	0.811	0.986
Right parahippocampal	EU Sample	-0.007	0.005	0.139	0.836
Right parsopercularis	EU Sample	-0.009	0.005	0.111	0.822
Right parsorbitalis	EU Sample	-0.006	0.005	0.222	0.836

Right parstriangularis	EU Sample	-0.007	0.006	0.227	0.836
Right pericalcarine	EU Sample	-0.002	0.005	0.655	0.928
Right postcentral	EU Sample	-0.007	0.005	0.184	0.836
Right posteriorcingulate	EU Sample	0.003	0.005	0.552	0.926
Right precentral	EU Sample	-0.012	0.007	0.080	0.822
Right precuneus	EU Sample	-0.003	0.005	0.578	0.926
Right rostralanteriorcingulate	EU Sample	0.005	0.006	0.378	0.926
Right rostralmiddlefrontal	EU Sample	-0.004	0.007	0.550	0.926
Right superiorfrontal	EU Sample	-0.002	0.006	0.775	0.986
Right superiorparietal	EU Sample	-0.005	0.007	0.401	0.926
Right superiortemporal	EU Sample	-0.012	0.005	0.024	0.493
Right supramarginal	EU Sample	-0.004	0.006	0.557	0.926
Right temporalpole	EU Sample	-0.018	0.008	0.021	0.493
Right transversetemporal	EU Sample	-0.007	0.005	0.180	0.836

**Brain Regions**	**Source of Results**	**β**	**SE**	***p***	***p*.FDR**
Left Banks of Superior Temporal Sulcus	Non-EU Sample	-0.005	0.006	0.340	0.514
Left caudalanteriorcingulate	Non-EU Sample	0.001	0.005	0.789	0.836
Left caudalmiddlefrontal	Non-EU Sample	0.005	0.008	0.547	0.648
Left cuneus	Non-EU Sample	0.000	0.006	0.962	0.966
Left entorhinal	Non-EU Sample	-0.012	0.008	0.141	0.351
Left frontalpole	Non-EU Sample	-0.016	0.007	0.027	0.139
Left fusiform	Non-EU Sample	-0.001	0.007	0.923	0.950
Left inferiorparietal	Non-EU Sample	-0.013	0.007	0.077	0.272
Left inferiortemporal	Non-EU Sample	-0.010	0.007	0.186	0.389
Left insula	Non-EU Sample	-0.008	0.008	0.322	0.508
Left isthmuscingulate	Non-EU Sample	-0.012	0.005	0.019	0.132
Left lateraloccipital	Non-EU Sample	-0.002	0.006	0.703	0.768
Left lateralorbitofrontal	Non-EU Sample	-0.010	0.008	0.200	0.406
Left lingual	Non-EU Sample	0.012	0.005	0.035	0.164

Left medialorbitofrontal	Non-EU Sample	0.005	0.008	0.498	0.622
Left middletemporal	Non-EU Sample	-0.010	0.007	0.148	0.351
Left paracentral	Non-EU Sample	-0.014	0.006	0.020	0.132
Left parahippocampal	Non-EU Sample	0.005	0.005	0.278	0.476
Left parsopercularis	Non-EU Sample	-0.013	0.007	0.063	0.264
Left parsorbitalis	Non-EU Sample	0.002	0.007	0.820	0.856
Left parstriangularis	Non-EU Sample	-0.007	0.007	0.337	0.514
Left pericalcarine	Non-EU Sample	0.007	0.006	0.268	0.476
Left postcentral	Non-EU Sample	-0.002	0.006	0.760	0.818
Left posteriorcingulate	Non-EU Sample	-0.008	0.005	0.127	0.337
Left precentral	Non-EU Sample	-0.007	0.007	0.375	0.533
Left precuneus	Non-EU Sample	-0.008	0.006	0.222	0.426
Left rostralanteriorcingulate	Non-EU Sample	0.004	0.008	0.650	0.732
Left rostralmiddlefrontal	Non-EU Sample	-0.008	0.008	0.318	0.508
Left superiorfrontal	Non-EU Sample	-0.006	0.007	0.423	0.566
Left superiorparietal	Non-EU Sample	-0.014	0.007	0.056	0.247
Left superiortemporal	Non-EU Sample	-0.005	0.006	0.422	0.566
Left supramarginal	Non-EU Sample	-0.011	0.008	0.128	0.337
Left temporalpole	Non-EU Sample	-0.012	0.009	0.178	0.384
Left transversetemporal	Non-EU Sample	-0.005	0.006	0.461	0.607
Mean cortical thickness in mm for left hemisphere	Non-EU Sample	-0.008	0.007	0.213	0.420
Mean cortical thickness in mm for right hemisphere	Non-EU Sample	-0.015	0.007	0.023	0.133
Mean cortical thickness in mm for whole brain	Non-EU Sample	-0.012	0.007	0.070	0.272
Right Banks of Superior Temporal Sulcus	Non-EU Sample	-0.006	0.005	0.236	0.441
Right caudalanteriorcingulate	Non-EU Sample	-0.009	0.005	0.073	0.272

Right caudalmiddlefrontal	Non-EU Sample	0.000	0.008	0.966	0.966
Right cuneus	Non-EU Sample	0.006	0.006	0.294	0.485
Right entorhinal	Non-EU Sample	-0.014	0.008	0.098	0.308
Right frontalpole	Non-EU Sample	-0.006	0.007	0.400	0.557
Right fusiform	Non-EU Sample	-0.004	0.007	0.566	0.659
Right inferiorparietal	Non-EU Sample	-0.017	0.007	0.020	0.132
Right inferiortemporal	Non-EU Sample	-0.010	0.007	0.147	0.351
Right insula	Non-EU Sample	-0.021	0.008	0.010	0.104
Right isthmuscingulate	Non-EU Sample	-0.015	0.005	0.002	0.084
Right lateraloccipital	Non-EU Sample	-0.008	0.006	0.173	0.384
Right lateralorbitofrontal	Non-EU Sample	-0.005	0.008	0.508	0.622
Right lingual	Non-EU Sample	0.008	0.006	0.128	0.337
Right medialorbitofrontal	Non-EU Sample	-0.004	0.008	0.583	0.668
Right middletemporal	Non-EU Sample	-0.006	0.007	0.363	0.533
Right paracentral	Non-EU Sample	-0.006	0.007	0.373	0.533
Right parahippocampal	Non-EU Sample	0.003	0.005	0.547	0.648
Right parsopercularis	Non-EU Sample	-0.007	0.006	0.253	0.461
Right parsorbitalis	Non-EU Sample	-0.018	0.007	0.007	0.104
Right parstriangularis	Non-EU Sample	-0.021	0.007	0.005	0.104
Right pericalcarine	Non-EU Sample	0.010	0.006	0.112	0.331
Right postcentral	Non-EU Sample	-0.017	0.007	0.010	0.104
Right posteriorcingulate	Non-EU Sample	-0.014	0.005	0.010	0.104
Right precentral	Non-EU Sample	-0.009	0.008	0.282	0.476
Right precuneus	Non-EU Sample	-0.003	0.006	0.678	0.752
Right rostralanteriorcingulate	Non-EU Sample	-0.012	0.007	0.100	0.308

Right rostralmiddlefrontal	Non-EU Sample	-0.005	0.008	0.502	0.622
Right superiorfrontal	Non-EU Sample	-0.010	0.007	0.173	0.384
Right superiorparietal	Non-EU Sample	-0.019	0.007	0.012	0.109
Right superiortemporal	Non-EU Sample	-0.015	0.007	0.026	0.139
Right supramarginal	Non-EU Sample	-0.028	0.007	0.000	0.007
Right temporalpole	Non-EU Sample	-0.015	0.009	0.100	0.308
Right transversetemporal	Non-EU Sample	-0.004	0.006	0.496	0.622

Supplementary Table S2. Summary of PGS-CRP Effects on Psychopathology

Exposure	Outcomes	Source of Results	β	SE	*p*	*p*.FDR
PGS-CRP	Depression	EU Sample	-0.115	0.085	0.176	0.371
PGS-CRP	Internalizing	EU Sample	-0.122	0.078	0.119	0.312
PGS-CRP	Externalizing	EU Sample	0.103	0.073	0.161	0.371
PGS-CRP	Depression	Non-EU Sample	0.224	0.095	0.019	0.031
PGS-CRP	Internalizing	Non-EU Sample	0.207	0.088	0.019	0.031
PGS-CRP	Externalizing	Non-EU Sample	0.242	0.083	0.003	0.012
PGS-CRP	Depression	Meta-analysis	0.051	0.170	0.761	0.809
PGS-CRP	Internalizing	Meta-analysis	0.040	0.164	0.809	0.809
PGS-CRP	Externalizing	Meta-analysis	0.167	0.069	0.016	0.048

Supplementary Table S3. Summary of Early-Life Infection Effects on Psychopathology

Exposure	Outcomes	Source of Results	β	SE	*p*	*p*.FDR
Early-Life Infection	Depression	EU Sample	0.312	0.182	0.087	0.312
Early-Life Infection	Internalizing	EU Sample	0.263	0.167	0.114	0.312
Early-Life Infection	Externalizing	EU Sample	0.382	0.157	0.015	0.104
Early-Life Infection	Depression	Non-EU Sample	0.224	0.096	0.019	0.031
Early-Life Infection	Internalizing	Non-EU Sample	0.207	0.088	0.019	0.031
Early-Life Infection	Externalizing	Non-EU Sample	0.242	0.083	0.004	0.012
Early-Life Infection	Depression	Meta-analysis	0.511	0.232	0.028	0.042
Early-Life Infection	Internalizing	Meta-analysis	0.608	0.363	0.094	0.094
Early-Life Infection	Externalizing	Meta-analysis	0.589	0.232	0.011	0.034

Supplementary Table S4. Summary of the Results from Mediation Pathways

**Direct Effect**	**Outcomes**	**β**	**SE**	***p***	***p*.FDR**
PGS-CRP	Mean Cortical Thickness Change	0.032	0.008	<0.001	<0.001
PGS-CRP	Depression	<0.001	0.011	0.966	0.966
PGS-CRP	Internalizing	-0.026	0.012	0.004	0.043
PGS-CRP	Externalizing	0.033	0.011	0.004	0.008
Mean Cortical Thickness Change	Depression	0.053	0.019	0.005	0.010
Mean Cortical Thickness Change	Internalizing	0.040	0.019	0.036	0.051
Mean Cortical Thickness Change	Externalizing	0.063	0.019	0.001	0.002
Indirect Effect		**β**	SE	*p*	*p*.FDR
Mean Cortical Thickness Change	Depression	0.002	0.001	0.022	0.036
Mean Cortical Thickness Change	Internalizing	0.001	0.001	0.054	0.072
Mean Cortical Thickness Change	Externalizing	0.002	0.001	0.008	0.014
Total Effect		**β**	SE	*p*	*p*.FDR
PGS-CRP	Depression	0.001	0.011	0.915	0.966
PGS-CRP	Internalizing	-0.024	0.012	0.037	0.051
PGS-CRP	Externalizing	0.035	0.011	0.002	0.005

Supplementary Table S5. Summary of Biological Annotation Results

Receptors	Names	r	*p*	*p*.FDR
$5HT_{1A}$	Serotonin 1A receptor	-0.160	0.069	0.224
$5HT_{1B}$	Serotonin 1B receptor	-0.044	0.376	0.533
$5HT_{2A}$	Serotonin 2A receptor	-0.052	0.277	0.428
$5HT_4$	Serotonin 4 receptor	-0.014	0.881	0.881
$5HT_6$	Serotonin 6 receptor	-0.250	0.010	0.085
5HTT	Serotonin transporter	-0.135	0.198	0.371
CB_1	Cannabinoid receptor type 1	-0.228	0.020	0.085
D_1	Dopamine receptor D1	-0.119	0.218	0.371
D_2	Dopamine receptor D2	-0.117	0.079	0.224
DAT	Dopamine transporter	-0.069	0.525	0.638
$GABA_A$	Gamma-Aminobutyric Acid A receptor	-0.274	0.010	0.085
H_3	Histamine H3 receptor	0.065	0.436	0.570
M_1	Muscarinic acetylcholine receptor M1	-0.127	0.208	0.371
$mGluR_5$	Metabotropic glutamate receptor 5	-0.253	0.020	0.085
MOR	Mu-opioid receptor	0.034	0.673	0.715
NET	Norepinephrine transporter	0.049	0.584	0.662
VAChT	Vesicular acetylcholine transporter	-0.147	0.139	0.338

Supplementary Table S6. Summary of PGS-CRP by Age Interaction Effects on Cortical Thickness Using Full Samples

Brain regions	β	SE	t.value	*p*	*p*.FDR
Left Banks of Superior Temporal Sulcus	-0.004	0.004	-1.150	0.250	0.414
Left caudalanteriorcingulate	0.000	0.003	0.123	0.902	0.924
Left caudalmiddlefrontal	-0.001	0.005	-0.177	0.859	0.911
Left cuneus	-0.001	0.003	-0.385	0.701	0.777
Left entorhinal	-0.014	0.005	-2.637	0.008	0.050
Left fusiform	-0.003	0.004	-0.731	0.465	0.579
Left inferiorparietal	-0.009	0.005	-1.907	0.057	0.177
Left inferiortemporal	-0.006	0.004	-1.415	0.157	0.319
Left isthmuscingulate	-0.008	0.003	-2.593	0.010	0.052
Left lateraloccipital	-0.002	0.004	-0.444	0.657	0.741
Left lateralorbitofrontal	-0.006	0.005	-1.359	0.174	0.326
Left lingual	0.003	0.003	0.877	0.380	0.519
Left medialorbitofrontal	0.001	0.005	0.158	0.874	0.913
Left middletemporal	-0.009	0.004	-2.029	0.042	0.159
Left parahippocampal	0.000	0.003	-0.111	0.911	0.924
Left paracentral	-0.009	0.004	-2.157	0.031	0.126
Left parsopercularis	-0.009	0.004	-2.146	0.032	0.126
Left parsorbitalis	-0.003	0.004	-0.810	0.418	0.540
Left parstriangularis	-0.005	0.005	-1.187	0.235	0.414
Left pericalcarine	0.004	0.004	1.055	0.291	0.444
Left postcentral	-0.003	0.004	-0.818	0.414	0.540
Left posteriorcingulate	-0.004	0.003	-1.149	0.251	0.414
Left precentral	-0.005	0.005	-0.988	0.323	0.468
Left precuneus	-0.006	0.004	-1.609	0.108	0.273
Left rostralanteriorcingulate	0.005	0.005	1.037	0.300	0.444
Left rostralmiddlefrontal	-0.004	0.005	-0.936	0.349	0.496
Left superiorfrontal	-0.005	0.004	-1.099	0.272	0.439
Left superiorparietal	-0.009	0.005	-1.900	0.057	0.177
Left superiortemporal	-0.008	0.004	-1.942	0.052	0.177
Left supramarginal	-0.006	0.005	-1.366	0.172	0.326
Left frontalpole	-0.005	0.005	-1.035	0.301	0.444
Left temporalpole	-0.009	0.006	-1.491	0.136	0.311
Left transversetemporal	-0.005	0.004	-1.351	0.177	0.326
Left insula	-0.008	0.005	-1.435	0.151	0.316
Right Banks of Superior Temporal Sulcus	-0.008	0.003	-2.375	0.018	0.083

Right caudalanteriorcingulate	-0.002	0.003	-0.668	0.504	0.606
Right caudalmiddlefrontal	-0.005	0.005	-1.075	0.283	0.444
Right cuneus	0.000	0.003	0.065	0.948	0.948
Right entorhinal	-0.016	0.005	-3.121	0.002	0.024
Right fusiform	-0.006	0.004	-1.516	0.129	0.306
Right inferiorparietal	-0.007	0.005	-1.444	0.149	0.316
Right inferiortemporal	-0.007	0.004	-1.725	0.085	0.231
Right isthmuscingulate	-0.008	0.003	-2.649	0.008	0.050
Right lateraloccipital	-0.003	0.003	-0.784	0.433	0.549
Right lateralorbitofrontal	-0.003	0.005	-0.568	0.570	0.663
Right lingual	0.002	0.003	0.715	0.475	0.581
Right medialorbitofrontal	-0.003	0.005	-0.616	0.538	0.637
Right middletemporal	-0.002	0.004	-0.489	0.625	0.715
Right parahippocampal	-0.003	0.003	-0.887	0.375	0.519
Right paracentral	-0.005	0.004	-1.178	0.239	0.414
Right parsopercularis	-0.009	0.004	-2.225	0.026	0.116
Right parsorbitalis	-0.012	0.004	-3.094	0.002	0.024
Right parstriangularis	-0.015	0.005	-3.317	0.001	0.022
Right pericalcarine	0.001	0.004	0.305	0.761	0.818
Right postcentral	-0.012	0.004	-3.141	0.002	0.024
Right posteriorcingulate	-0.006	0.004	-1.738	0.082	0.231
Right precentral	-0.009	0.005	-1.921	0.055	0.177
Right precuneus	-0.006	0.004	-1.440	0.150	0.316
Right rostralanteriorcingulate	-0.004	0.005	-0.836	0.403	0.540
Right rostralmiddlefrontal	-0.008	0.005	-1.587	0.113	0.275
Right superiorfrontal	-0.006	0.005	-1.343	0.179	0.326
Right superiorparietal	-0.013	0.005	-2.841	0.005	0.038
Right superiortemporal	-0.014	0.004	-3.339	0.001	0.022
Right supramarginal	-0.014	0.005	-3.046	0.002	0.024
Right frontalpole	0.002	0.005	0.341	0.733	0.801
Right temporalpole	-0.015	0.006	-2.674	0.008	0.050
Right transversetemporal	-0.006	0.004	-1.661	0.097	0.255
Right insula	-0.018	0.005	-3.460	0.001	0.022
Mean cortical thickness in mm for left hemisphere	-0.007	0.004	-1.793	0.073	0.216
Mean cortical thickness in mm for right hemisphere	-0.012	0.004	-2.820	0.005	0.038
Mean cortical thickness in mm for whole brain	-0.010	0.004	-2.383	0.017	0.083

Supplementary Table S7. Summary of PGS-CRP Effects on Psychopathology Using Full Sample

**Exposure**	**Outcomes**	**Source of Results**	**β**	**SE**	***p***	***p*.FDR**
PGS-CRP	Depression	Full Sample	0.070	0.062	0.258	0.362
PGS-CRP	Internalizing	Full Sample	0.064	0.057	0.263	0.362
PGS-CRP	Externalizing	Full Sample	0.167	0.054	0.002	0.007

Supplementary Table S8 . Summary of Early-Life Infection Effects on Psychopathology Using Full Sample

**Exposure**	**Outcomes**	**Source of Results**	**β**	**SE**	***p***	***p*.FDR**
Early-Life Infection	Depression	Full Sample	0.431	0.147	0.003	0.010
Early-Life Infection	Internalizing	Full Sample	0.486	0.134	0.000	0.001
Early-Life Infection	Externalizing	Full Sample	0.541	0.126	0.000	<0.001

Supplementary Table S9. Sensitivity Analysis examining Associations between Early-Life Infection (defined as ≥3 days of illness during infancy) and Cortical Thickness.

Exposure	Brain Region	Source of Results	β	SE	*p*	*p*.FDR
Early-Life Infection	Left Banks of Superior Temporal Sulcus	Meta-analysis	-0.051	0.234	0.826	0.952
Early-Life Infection	Left caudalanteriorcingulate	Meta-analysis	-0.198	0.174	0.254	0.952
Early-Life Infection	Left caudalmiddlefrontal	Meta-analysis	-0.045	0.145	0.755	0.952
Early-Life Infection	Left cuneus	Meta-analysis	-0.006	0.105	0.955	0.977
Early-Life Infection	Left entorhinal	Meta-analysis	0.047	0.273	0.863	0.952
Early-Life Infection	Left frontalpole	Meta-analysis	-0.081	0.135	0.548	0.952
Early-Life Infection	Left fusiform	Meta-analysis	0.035	0.173	0.841	0.952
Early-Life Infection	Left inferiorparietal	Meta-analysis	-0.160	0.398	0.687	0.952
Early-Life Infection	Left inferiortemporal	Meta-analysis	-0.179	0.287	0.532	0.952
Early-Life Infection	Left insula	Meta-analysis	0.008	0.163	0.961	0.977
Early-Life Infection	Left isthmuscingulate	Meta-analysis	0.124	0.131	0.342	0.952
Early-Life Infection	Left lateraloccipital	Meta-analysis	-0.071	0.315	0.823	0.952
Early-Life Infection	Left lateralorbitofrontal	Meta-analysis	-0.075	0.145	0.603	0.952
Early-Life Infection	Left lingual	Meta-analysis	-0.069	0.223	0.758	0.952
Early-Life Infection	Left medialorbitofrontal	Meta-analysis	0.075	0.152	0.623	0.952
Early-Life Infection	Left middletemporal	Meta-analysis	-0.438	0.345	0.205	0.952
Early-Life Infection	Left paracentral	Meta-analysis	-0.176	0.277	0.524	0.952

Early-Life Infection	Left parahippocampal	Meta-analysis	-0.051	0.191	0.789	0.952
Early-Life Infection	Left parsopercularis	Meta-analysis	-0.181	0.131	0.168	0.952
Early-Life Infection	Left parsorbitalis	Meta-analysis	0.040	0.124	0.748	0.952
Early-Life Infection	Left parstriangularis	Meta-analysis	-0.291	0.207	0.160	0.952
Early-Life Infection	Left pericalcarine	Meta-analysis	-0.010	0.215	0.963	0.977
Early-Life Infection	Left postcentral	Meta-analysis	-0.183	0.199	0.358	0.952
Early-Life Infection	Left posteriorcingulate	Meta-analysis	-0.087	0.102	0.397	0.952
Early-Life Infection	Left precentral	Meta-analysis	-0.160	0.195	0.414	0.952
Early-Life Infection	Left precuneus	Meta-analysis	-0.038	0.119	0.747	0.952
Early-Life Infection	Left rostralanteriorcingulate	Meta-analysis	-0.007	0.150	0.963	0.977
Early-Life Infection	Left rostralmiddlefrontal	Meta-analysis	-0.112	0.175	0.523	0.952
Early-Life Infection	Left superiorfrontal	Meta-analysis	-0.124	0.132	0.347	0.952
Early-Life Infection	Left superiorparietal	Meta-analysis	-0.253	0.274	0.356	0.952
Early-Life Infection	Left superiortemporal	Meta-analysis	-0.184	0.191	0.335	0.952
Early-Life Infection	Left supramarginal	Meta-analysis	-0.194	0.173	0.263	0.952
Early-Life Infection	Left temporalpole	Meta-analysis	-0.232	0.430	0.588	0.952
Early-Life Infection	Left transversetemporal	Meta-analysis	-0.029	0.117	0.804	0.952
Early-Life Infection	Mean cortical thickness in mm for left hemisphere	Meta-analysis	-0.235	0.316	0.458	0.952
Early-Life Infection	Mean cortical thickness in mm for right hemisphere	Meta-analysis	-0.269	0.284	0.344	0.952
Early-Life Infection	Mean cortical thickness in mm for whole brain	Meta-analysis	-0.259	0.304	0.395	0.952

Early-Life Infection	Right Banks of Superior Temporal Sulcus	Meta-analysis	-0.019	0.103	0.855	0.952
Early-Life Infection	Right caudalanteriorcingulate	Meta-analysis	-0.030	0.141	0.831	0.952
Early-Life Infection	Right caudalmiddlefrontal	Meta-analysis	-0.370	0.319	0.247	0.952
Early-Life Infection	Right cuneus	Meta-analysis	-0.045	0.220	0.837	0.952
Early-Life Infection	Right entorhinal	Meta-analysis	0.003	0.157	0.985	0.985
Early-Life Infection	Right frontalpole	Meta-analysis	-0.127	0.139	0.361	0.952
Early-Life Infection	Right fusiform	Meta-analysis	-0.194	0.129	0.131	0.952
Early-Life Infection	Right inferiorparietal	Meta-analysis	-0.264	0.332	0.427	0.952
Early-Life Infection	Right inferiortemporal	Meta-analysis	0.105	0.130	0.422	0.952
Early-Life Infection	Right insula	Meta-analysis	0.077	0.163	0.637	0.952
Early-Life Infection	Right isthmuscingulate	Meta-analysis	0.101	0.098	0.303	0.952
Early-Life Infection	Right lateraloccipital	Meta-analysis	-0.223	0.194	0.250	0.952
Early-Life Infection	Right lateralorbitofrontal	Meta-analysis	-0.241	0.154	0.118	0.952
Early-Life Infection	Right lingual	Meta-analysis	-0.027	0.255	0.916	0.977
Early-Life Infection	Right medialorbitofrontal	Meta-analysis	-0.141	0.149	0.343	0.952
Early-Life Infection	Right middletemporal	Meta-analysis	-0.161	0.296	0.587	0.952
Early-Life Infection	Right paracentral	Meta-analysis	-0.151	0.159	0.342	0.952
Early-Life Infection	Right parahippocampal	Meta-analysis	-0.155	0.105	0.139	0.952
Early-Life Infection	Right parsopercularis	Meta-analysis	-0.111	0.178	0.532	0.952
Early-Life Infection	Right parsorbitalis	Meta-analysis	-0.024	0.121	0.842	0.952

Early-Life Infection	Right parstriangularis	Meta-analysis	-0.192	0.246	0.436	0.952
Early-Life Infection	Right pericalcarine	Meta-analysis	-0.203	0.299	0.496	0.952
Early-Life Infection	Right postcentral	Meta-analysis	-0.067	0.244	0.785	0.952
Early-Life Infection	Right posteriorcingulate	Meta-analysis	0.017	0.108	0.872	0.952
Early-Life Infection	Right precentral	Meta-analysis	-0.255	0.149	0.087	0.952
Early-Life Infection	Right precuneus	Meta-analysis	-0.092	0.216	0.670	0.952
Early-Life Infection	Right rostralanteriorcingulate	Meta-analysis	0.151	0.138	0.274	0.952
Early-Life Infection	Right rostralmiddlefrontal	Meta-analysis	-0.299	0.215	0.163	0.952
Early-Life Infection	Right superiorfrontal	Meta-analysis	-0.291	0.165	0.077	0.952
Early-Life Infection	Right superiorparietal	Meta-analysis	-0.119	0.239	0.619	0.952
Early-Life Infection	Right superiortemporal	Meta-analysis	-0.058	0.180	0.748	0.952
Early-Life Infection	Right supramarginal	Meta-analysis	-0.043	0.199	0.830	0.952
Early-Life Infection	Right temporalpole	Meta-analysis	-0.098	0.173	0.570	0.952
Early-Life Infection	Right transversetemporal	Meta-analysis	-0.116	0.131	0.375	0.952

Results were consistent with the primary analysis, showing no significant association (p.FDR > 0.05) between early-life infections and cortical thickness in any brain region. This indicates that the meta-analysis model was robust to different operationalizations of infection history.

Supplementary Table S10. Sensitivity Analysis examining Associations between Early-Life Infection (defined as ≥3 days of illness during infancy)and Psychopathology Outcomes.

Exposure	Outcomes	Source of Results	β	SE	*p*	*p*.FDR
Early-Life Infection	Depression	Meta-analysis	0.408	0.175	0.019	0.019
Early-Life Infection	Externalizing	Meta-analysis	0.365	0.150	0.015	0.019
Early-Life Infection	Internalizing	Meta-analysis	0.416	0.160	0.009	0.019

A stricter infection threshold (≥3 sick days) revealed significant associations with depression, externalizing, and internalizing symptoms (p.FDR < 0.05), whereas the primary analysis identified only depression and externalizing effects, supporting robustness across infection definitions.

Supplementary Table S11. Sensitivity Analysis of the Effects of Early-Life Infection and PGS-CRP on Youth-Reported (BPM-Y) Psychopathology Outcomes.

Exposure	Outcomes	Source of Results	β	SE	*p*	*p*.FDR
Early-Life Infection	Externalizing (Youth T)	Meta-analysis	0.535	0.195	0.006	0.018
Early-Life Infection	Internalizing (Youth T)	Meta-analysis	0.361	0.197	0.067	0.101
Early-Life Infection	Total Problems (Youth T)	Meta-analysis	0.203	0.194	0.293	0.293
PGS-CRP	Internalizing (Youth T)	Meta-analysis	0.132	0.181	0.463	0.723
PGS-CRP	Total Problems (Youth T)	Meta-analysis	0.185	0.264	0.482	0.723
PGS-CRP	Externalizing (Youth T)	Meta-analysis	0.097	0.275	0.723	0.723

Early-life infection was associated with higher youth-reported externalizing symptoms (β = 0.54, p.FDR = 0.018), with trend-level effects for internalizing (β = 0.36, p.FDR = 0.10) and total problems (β = 0.20, p.FDR = 0.29). In contrast, polygenic scores for CRP (PGS-CRP) were not significantly related to any youth psychopathology outcomes (all p.FDR > 0.70).

Supplementary Table S12. Sensitivity Analysis of the Interaction between PGS-CRP and Age on Cortical Brain Thickness, adjusting only for Genetic Principal Components.

Brain Region	Source of Results	β	SE	*p*	*p*.FDR
Right insula	Meta-analysis	-0.020	0.005	<0.001	<0.001
Right temporalpole	Meta-analysis	-0.018	0.006	0.001	0.001
Right entorhinal	Meta-analysis	-0.015	0.005	0.003	0.003
Right parstriangularis	Meta-analysis	-0.015	0.012	0.214	0.214
Right supramarginal	Meta-analysis	-0.015	0.016	0.365	0.365
Left entorhinal	Meta-analysis	-0.015	0.005	0.004	0.004
Right superiortemporal	Meta-analysis	-0.014	0.007	0.036	0.036
Left temporalpole	Meta-analysis	-0.014	0.009	0.105	0.105
Right parsorbitalis	Meta-analysis	-0.013	0.008	0.091	0.091
Right postcentral	Meta-analysis	-0.012	0.008	0.109	0.109
Mean cortical thickness in mm for right hemisphere	Meta-analysis	-0.012	0.010	0.251	0.251
Right superiorparietal	Meta-analysis	-0.011	0.010	0.264	0.264
Right inferiortemporal	Meta-analysis	-0.010	0.009	0.256	0.256
Mean cortical thickness in mm for whole brain	Meta-analysis	-0.009	0.009	0.283	0.283
Right precentral	Meta-analysis	-0.009	0.005	0.053	0.053
Right parsopercularis	Meta-analysis	-0.009	0.004	0.018	0.018
Left inferiorparietal	Meta-analysis	-0.009	0.009	0.302	0.302
Left parsopercularis	Meta-analysis	-0.009	0.004	0.049	0.049
Left supramarginal	Meta-analysis	-0.008	0.011	0.470	0.470
Right superiorfrontal	Meta-analysis	-0.008	0.008	0.320	0.320
Left middletemporal	Meta-analysis	-0.008	0.005	0.148	0.148
Left superiortemporal	Meta-analysis	-0.008	0.004	0.044	0.044
Left inferiortemporal	Meta-analysis	-0.007	0.008	0.389	0.389
Mean cortical thickness in mm for left hemisphere	Meta-analysis	-0.007	0.007	0.337	0.337
Left superiorparietal	Meta-analysis	-0.007	0.010	0.467	0.467
Right rostralmiddlefrontal	Meta-analysis	-0.007	0.007	0.355	0.355
Left isthmuscingulate	Meta-analysis	-0.006	0.003	0.053	0.053

Left superiorfrontal	Meta-analysis	-0.006	0.006	0.314	0.314
Left insula	Meta-analysis	-0.006	0.005	0.252	0.252
Right isthmuscingulate	Meta-analysis	-0.006	0.006	0.311	0.311
Right Banks of Superior Temporal Sulcus	Meta-analysis	-0.006	0.003	0.071	0.071
Left paracentral	Meta-analysis	-0.006	0.009	0.526	0.526
Left lateralorbitofrontal	Meta-analysis	-0.006	0.006	0.374	0.374
Right fusiform	Meta-analysis	-0.005	0.004	0.190	0.190
Right transversetemporal	Meta-analysis	-0.005	0.004	0.159	0.159
Left parsorbitalis	Meta-analysis	-0.005	0.004	0.185	0.185
Right inferiorparietal	Meta-analysis	-0.005	0.016	0.738	0.738
Right caudalmiddlefrontal	Meta-analysis	-0.005	0.005	0.283	0.283
Left frontalpole	Meta-analysis	-0.005	0.012	0.699	0.699
Left precuneus	Meta-analysis	-0.005	0.008	0.564	0.564
Right middletemporal	Meta-analysis	-0.004	0.009	0.640	0.640
Right lateralorbitofrontal	Meta-analysis	-0.004	0.008	0.568	0.568
Right posteriorcingulate	Meta-analysis	-0.004	0.007	0.554	0.554
Right paracentral	Meta-analysis	-0.004	0.004	0.360	0.360
Right medialorbitofrontal	Meta-analysis	-0.003	0.005	0.524	0.524
Left rostralmiddlefrontal	Meta-analysis	-0.003	0.007	0.644	0.644
Left lingual	Meta-analysis	0.003	0.005	0.551	0.551
Left transversetemporal	Meta-analysis	-0.003	0.004	0.388	0.388
Left rostralanteriorcingulate	Meta-analysis	0.003	0.005	0.554	0.554
Left pericalcarine	Meta-analysis	0.003	0.004	0.406	0.406
Right frontalpole	Meta-analysis	0.003	0.006	0.620	0.620
Left precentral	Meta-analysis	-0.003	0.005	0.565	0.565
Right rostralanteriorcingulate	Meta-analysis	-0.003	0.010	0.767	0.767
Right precuneus	Meta-analysis	-0.003	0.004	0.527	0.527
Left postcentral	Meta-analysis	-0.003	0.004	0.462	0.462
Left fusiform	Meta-analysis	-0.003	0.004	0.538	0.538
Left Banks of Superior Temporal Sulcus	Meta-analysis	-0.002	0.007	0.728	0.728

Right cuneus	Meta-analysis	0.002	0.003	0.486	0.486
Right pericalcarine	Meta-analysis	0.002	0.004	0.600	0.600
Left posteriorcingulate	Meta-analysis	-0.002	0.005	0.683	0.683
Left parstriangularis	Meta-analysis	-0.002	0.004	0.621	0.621
Left parahippocampal	Meta-analysis	0.002	0.004	0.579	0.579
Left lateraloccipital	Meta-analysis	-0.002	0.005	0.668	0.668
Right lingual	Meta-analysis	0.002	0.003	0.555	0.555
Right parahippocampal	Meta-analysis	-0.002	0.004	0.648	0.648
Left medialorbitofrontal	Meta-analysis	0.001	0.005	0.817	0.817
Right lateraloccipital	Meta-analysis	-0.001	0.007	0.866	0.866
Right caudalanteriorcingulate	Meta-analysis	-0.001	0.004	0.772	0.772
Left caudalanteriorcingulate	Meta-analysis	0.001	0.003	0.836	0.836
Left caudalmiddlefrontal	Meta-analysis	-0.000	0.005	0.968	0.968
Left cuneus	Meta-analysis	-0.000	0.003	0.995	0.995

Results were consistent with the primary analyses, indicating that the interaction between PGS-CRP and Age on cortical brain thickness was robust to adjustment using only ancestry-related covariates. Eight additional subregions surpassed the FDR threshold (p.FDR < 0.05), suggesting broader cortical involvement than observed in the primary analyses.

Supplementary Table S13. Sensitivity Analysis of the Effect of PGS-CRP on Psychopathology Outcomes, adjusting only for Genetic Principal Components.

**Outcomes**	**Source of Results**	**β**	**SE**	***p***	***p*.FDR**
Externalizing	Meta-analysis	0.206	0.084	0.015	0.044
Internalizing	Meta-analysis	0.071	0.196	0.716	0.798
Depression	Meta-analysis	0.047	0.185	0.798	0.798

The sensitivity analysis adjusting only for genetic principal components yielded a comparable pattern of results, with a similarly sized and directionally consistent association between PGS-CRP and externalizing symptoms ($\beta = 0.206$, p.FDR = 0.044). Effects for internalizing and depression remained non-significant. These findings indicate that the observed relationship between genetic predisposition to systemic inflammation and externalizing psychopathology is robust and not dependent on the inclusion of additional covariates, further supporting the primary results.

Supplementary Table S14. Demographic, exposure, and outcome variables in ancestry-stratified samples (EU and Non-EU) and full meta-analysis sample for in Year 0 and Year 2.

**EU Sample (Sample 1)**	**Year 0 (n = 6,336)**	**Year 2 (n = 4,617)**
Mean cortical thickness (mean [SD][4])	2.74 (0.08)	2.70 (0.08)
Depression (mean [SD])	53.60 (5.66)	53.94 (6.02)
Internalizing (mean [SD])	48.70 (10.45)	48.30 (10.33)
Externalizing (mean [SD])	45.42 (10.01)	44.47 (9.65)
Polygenic score for CRP[5] (mean [SD])	−0.18 (0.93)	−0.18 (0.93)
Age (years)	9.92 (0.63)	11.96 (0.65)
Sex at birth: female (%)	2,982 (47.1%)	2,080 (45.1%)
Body mass index (mean [SD])	17.92 (3.31)	19.69 (4.01)
Race/ethnicity (%)		
White	5,807 (91.7%)	4,225 (91.9%)
Black	3 (0.0%)	2 (0.0%)
Hispanic	270 (4.3%)	182 (4.0%)
Asian	4 (0.1%)	2 (0.0%)
Multiracial/Other	251 (4.0%)	185 (4.0%)
Parental education (%)		
Less than high school	37 (0.6%)	31 (0.7%)
High school diploma	221 (3.5%)	149 (3.2%)
Some college	1,208 (19.1%)	864 (18.8%)
Bachelor’s degree	1,967 (31.1%)	1,445 (31.5%)
Postgraduate degree	2,892 (45.7%)	2,100 (45.8%)
Household income (%)		
< $50,000	780 (12.9%)	566 (12.9%)
$50,000–99,999	1,856 (30.8%)	1,404 (32.0%)
≥ $100,000	3,393 (56.3%)	2,424 (55.2%)
**Non-EU Sample (Sample 2)**	**Year 0 (n = 4,878)**	**Year 2 (n = 3,206)**
Mean cortical thickness (mean [SD])	2.70 (0.08)	2.66 (0.08)
Depression (mean [SD])	53.61 (5.80)	53.55 (5.83)
Internalizing (mean [SD])	48.19 (10.91)	46.96 (10.76)

[4] SD: Standard deviation

[5] CRP: C-reactive protein

Externalizing (mean [SD])	46.26 (10.74)	44.81 (10.18)
Polygenic score for CRP (mean [SD])	0.25 (1.04)	0.24 (1.03)
Age (years)	9.90 (0.62)	11.94 (0.66)
Sex at birth: female (%)	2,347 (48.1%)	1,511 (47.1%)
Body mass index (mean [SD])	19.78 (4.46)	21.66 (4.80)
Race/ethnicity (%)		
White	117 (2.4%)	81 (2.6%)
Black	1,667 (34.2%)	1,048 (33.1%)
Hispanic	1,947 (39.9%)	1,277 (40.3%)
Asian	209 (4.3%)	128 (4.0%)
Multiracial/Other	973 (19.2%)	631 (19.9%)
Parental education (%)		
Less than high school	476 (9.9%)	303 (9.7%)
High school diploma	806 (16.7%)	493 (15.8%)
Some college	1,702 (35.3%)	1,122 (35.9%)
Bachelor's degree	899 (18.7%)	606 (19.4%)
Postgraduate degree	973 (19.4%)	600 (19.2%)
Household income (%)		
< $50,000	2,228 (52.4%)	1,447 (52.1%)
$50,000–99,999	1,074 (25.3%)	743 (26.8%)
≥ $100,000	946 (22.3%)	585 (21.1%)
**Full Meta-Analysis Sample**	**Year 0 (n = 11,214)**	**Year 2 (n = 7,823)**
Mean cortical thickness (mean [SD])	2.72 (0.08)	2.69 (0.08)
Depression (mean [SD])	53.60 (5.72)	53.78 (5.95)
Internalizing (mean [SD])	48.48 (10.66)	47.75 (10.53)
Externalizing (mean [SD])	45.77 (10.34)	44.63 (9.87)
Polygenic score for CRP (mean [SD])	0.00 (1.00)	−0.01 (1.00)
Early-life infection: Yes (%)	1,710 (16.7%)	1,218 (16.9%)
Age (years)	9.91 (0.63)	11.95 (0.65)
Sex at birth: female (%)	5,329 (47.5%)	3,591 (45.9%)
Body mass index (mean [SD])	18.73 (3.96)	20.49 (4.45)
Race/ethnicity (%)		
White	5,924 (52.8%)	4,306 (55.5%)
Black	1,670 (14.9%)	1,050 (13.5%)

Hispanic	2,217 (19.8%)	1,459 (18.8%)
Asian	213 (1.9%)	130 (1.7%)
Multiracial/Other	1,188 (10.6%)	816 (10.5%)
Parental education (%)		
Less than high school	513 (4.6%)	334 (4.3%)
High school diploma	1,027 (9.2%)	642 (8.3%)
Some college	2,910 (26.1%)	1,986 (25.7%)
Bachelor's degree	2,866 (25.7%)	2,051 (26.6%)
Postgraduate degree	3,829 (34.4%)	2,700 (35.0%)
Household income (%)		
< $50,000	3,008 (29.3%)	2,013 (28.1%)
$50,000–99,999	2,930 (28.5%)	2,147 (29.9%)
≥ $100,000	4,339 (42.2%)	3,009 (42.0%)

Supplementary Table S15. Summary of Missing Data for Exposures, Outcomes, and Covariates in Baseline (Y0) and 2-Year Follow-up (Y2) Cohorts.

	Participants in 2-year follow-up (Y0 and Y2)				Participants not in 2-year follow-up (Only Y0)			
**Variable**	**n completed Y0 and Y2**	**n missing**	**n present**	**% missing**	**n completed only Y0**	**n missing**	**n present**	**% missing**
**Mean Cortical Thickness**	7,746	0	7,746	0.0	3,468	0	3,468	0.0
**CBCL[6] Depression (T-value)**	7,746	1	7,745	0.0	3,468	6	3,462	0.2
**CBCL Internalizing (T-value)**	7,746	1	7,745	0.0	3,468	6	3,462	0.2
**CBCL Externalizing (T-value)**	7,746	1	7,745	0.0	3,468	6	3,462	0.2
**Polygenic Score (CRP)**	7,746	0	7,746	0.0	3,468	0	3,468	0.0
**Any Sick Days**	7,746	612	7,134	7.9	3,468	349	3,119	10.1
**Age (years)**	7,746	0	7,746	0.0	3,468	0	3,468	0.0
**Sex**	7,746	1	7,745	0.0	3,468	2	3,466	0.1
**BMI**	7,746	38	7,708	0.5	3,468	43	3,425	1.2
**Parental Education**	7,746	48	7,698	0.6	3,468	21	3,447	0.6
**Household Income**	7,746	590	7,156	7.6	3,468	347	3,121	10.0
**Race/Ethnicity**	7,746	0	7,746	0.0	3,468	2	3,466	0.1

[6] CBCL = Child Behavior Checklist

Supplementary Table S16. Summary of Attrition between Baseline (Y0) and 2-Year Follow-up (Y2) Cohorts.

Variable	Level	Completed Y0 and Y2	Completed only Y0	*p*	SMD[7]
**Mean Cortical Thickness**		2.72 (0.08)	2.72 (0.09)	0.338	0.02
**CBCL Depression (T)**		53.55 (5.65)	53.72 (5.87)	0.149	-0.03
**CBCL Internalizing (T)**		48.49 (10.55)	48.47 (10.90)	0.924	0.00
**CBCL Externalizing (T)**		45.73 (10.30)	45.85 (10.44)	0.582	-0.01
**Polygenic Score (CRP)**		-0.01 (0.99)	0.03 (1.02)	0.094	-0.03
**Any Sick Days**				0.295	0.01
	No	5926 (83.1%)	2617 (83.9%)	0.295	0.01
	Yes	1208 (16.9%)	502 (16.1%)		
**Age (years)**		9.89 (0.62)	9.96 (0.64)	<0.001	-0.11
**Sex**				<0.001	0.05
	Male	4194 (54.2%)	1688 (48.7%)	<0.001	0.05
	Female	3551 (45.8%)	1778 (51.3%)		
**BMI**		18.63 (3.87)	18.94 (4.16)	<0.001	-0.08
**Parental Education**				<0.001	0.06
	< HS Diploma	334 (4.3%)	179 (5.2%)	<0.001	0.06
	HS Diploma/GED	640 (8.3%)	387 (11.2%)		
	Some College	1985 (25.8%)	925 (26.8%)		
	Bachelor	2047 (26.6%)	819 (23.8%)		
	Post Graduate Degree	2692 (35.0%)	1137 (33.0%)		
**Household Income**				<0.001	0.05
	[<50K]	2011 (28.1%)	997 (31.9%)	<0.001	0.05
	[>=50K & <100K]	2145 (30.0%)	785 (25.2%)		
	[>=100K]	3000 (41.9%)	1339 (42.9%)		
**Race/Ethnicity**				<0.001	0.09
	White/Not Hispanic	4298 (55.5%)	1626 (46.9%)	<0.001	0.09
	Black/Not Hispanic	1048 (13.5%)	622 (17.9%)		
	Hispanic	1457 (18.8%)	760 (21.9%)		
	Asian	129 (1.7%)	84 (2.4%)		
	Multiracial	814 (10.5%)	374 (10.8%)		

Although several baseline variables differed statistically between participants who completed both Y0 and Y2 assessments and those who completed only Y0 (e.g., age, sex, socioeconomic factors; all $p < 0.001$), all standardized mean differences were small (≤ 0.11), indicating negligible effect sizes. Overall, attrition between Y0 and Y2 was minimal and unlikely to bias study findings.

[7] SMD = Standard Mean Difference
