# Supplementary material for: Polygenic score for C-reactive protein is linked to faster cortical thinning and psychopathology risk in adolescents
Source: Nat Ment Health. 2026 Feb 16;4(3):427–38. doi: 10.1038/s44220-026-00585-w (PMC12975513; doi:10.1038/s44220-026-00585-w)
Supplement: Supplementary file 2 — Reporting Summary [file 44220_2026_585_MOESM2_ESM.pdf]

nature portfolio

Corresponding author(s): Haixia Zheng

Last updated by author(s): Dec 5, 2025

Reporting Summary

Nature Portfolio wishes to improve the reproducibility of the work that we publish. This form provides structure for consistency and transparency in reporting. For further information on Nature Portfolio policies, see our Editorial Policies and the Editorial Policy Checklist.

Statistics

For all statistical analyses, confirm that the following items are present in the figure legend, table legend, main text, or Methods section.

n/a	Confirmed	
☐	☒	The exact sample size (n) for each experimental group/condition, given as a discrete number and unit of measurement
☐	☒	A statement on whether measurements were taken from distinct samples or whether the same sample was measured repeatedly
☐	☒	The statistical test(s) used AND whether they are one- or two-sided<br>*Only common tests should be described solely by name; describe more complex techniques in the Methods section.*
☐	☒	A description of all covariates tested
☐	☒	A description of any assumptions or corrections, such as tests of normality and adjustment for multiple comparisons
☐	☒	A full description of the statistical parameters including central tendency (e.g. means) or other basic estimates (e.g. regression coefficient) AND variation (e.g. standard deviation) or associated estimates of uncertainty (e.g. confidence intervals)
☒	☐	For null hypothesis testing, the test statistic (e.g. F, t, r) with confidence intervals, effect sizes, degrees of freedom and P value noted<br>*Give P values as exact values whenever suitable.*
☐	☒	For Bayesian analysis, information on the choice of priors and Markov chain Monte Carlo settings
☐	☒	For hierarchical and complex designs, identification of the appropriate level for tests and full reporting of outcomes
☐	☒	Estimates of effect sizes (e.g. Cohen's d, Pearson's r), indicating how they were calculated

Our web collection on statistics for biologists contains articles on many of the points above.

Software and code

Policy information about availability of computer code

Data collection	This study used data from the Adolescent Brain Cognitive Development (ABCD) Study, curated release 5.1. These data are publicly available through the National Institute of Mental Health Data Archive (NDA) under the ABCD collection (https://nda.nih.gov/abcd). Access requires completion of the NDA Data Use Certification.
Data analysis	No custom algorithms were developed for this study. Analyses were performed using publicly available software, including PLINK 2.0 and PRS-CS for genetic analyses; FreeSurfer v7.1.1 for neuroimaging processing; and R (version 4.4.1) with the packages nlme (v3.1-168), metafor (v4.8-0), and lavaan (v0.6-19) for statistical modelling and mediation analyses. Neurobiological annotation was conducted using the Neuromaps toolbox. All software used in this study is freely accessible.

For manuscripts utilizing custom algorithms or software that are central to the research but not yet described in published literature, software must be made available to editors and reviewers. We strongly encourage code deposition in a community repository (e.g. GitHub). See the Nature Portfolio guidelines for submitting code & software for further information.

Data

Policy information about availability of data

All manuscripts must include a data availability statement. This statement should provide the following information, where applicable:

- Accession codes, unique identifiers, or web links for publicly available datasets
- A description of any restrictions on data availability
- For clinical datasets or third party data, please ensure that the statement adheres to our policy

This study used data from the Adolescent Brain Cognitive Development (ABCD) Study, curated release 5.1. These data are publicly available through the National Institute of Mental Health Data Archive (NDA) under the ABCD collection (https://nda.nih.gov/abcd). Access requires completion of the NDA Data Use Certification.

Research involving human participants, their data, or biological material

Policy information about studies with human participants or human data. See also policy information about sex, gender (identity/presentation), and sexual orientation and race, ethnicity and racism.

Reporting on sex and gender	Sex at birth (male/female) was collected via parent report in the ABCD Study and included as a fixed-effect covariate in all analyses. Sex was considered in study design and analyses because it is known to influence neurodevelopment and health outcomes. Gender identity was not collected or analyzed in this dataset. Findings apply to participants of all sexes combined, as no sex-stratified analyses were conducted; the lack of sex-specific models reflects the study's focus on genetic and neurodevelopmental effects rather than sex differences. Disaggregated sex data are available in the ABCD dataset, and all data were collected with parental consent and child assent.
Reporting on race, ethnicity, or other socially relevant groupings	Race/ethnicity, parental education, and household income were included as fixed-effect covariates in analyses. Parental education and household income, which were reported by the child's guardian, are key indicators of socioeconomic status, which is known to be associated with neurodevelopmental trajectories and psychopathology.
Population characteristics	Age, body mass index (BMI), and the first ten principal components of ancestry were included as fixed-effect covariate in analyses. These variables are well-established correlates of both systemic inflammation and/or brain structure, and therefore help reduce residual confounding.
Recruitment	Recruitment was conducted primarily through public and private elementary schools using a probability sampling approach stratified by age, sex, race/ethnicity, socioeconomic status, and urbanicity, with targeted oversampling of underrepresented groups (e.g., African American, Hispanic, and rural youth) to better reflect the U.S. population. The participating schools were drawn from each site's catchment area, collectively encompassing ~20% of the U.S. population of 9–10-year-olds. Approximately 9.6% of contacted families enrolled, yielding a cohort broadly representative of the sociodemographic composition of U.S. children. Enrollment demographics were monitored throughout recruitment, and later school samples were dynamically adjusted to correct deviations from target demographics. Approximately half of the sample was enriched for children showing early signs of externalizing or internalizing symptoms to ensure sufficient power for studying developmental risk trajectories.
Ethics oversight	Institutional Review Board (IRB)

Note that full information on the approval of the study protocol must also be provided in the manuscript.

Field-specific reporting

Please select the one below that is the best fit for your research. If you are not sure, read the appropriate sections before making your selection.

☐ Life sciences ☒ Behavioural & social sciences ☐ Ecological, evolutionary & environmental sciences

For a reference copy of the document with all sections, see nature.com/documents/nr-reporting-summary-flat.pdf

Behavioural & social sciences study design

All studies must disclose on these points even when the disclosure is negative.

Study description	Quantitative, observational, longitudinal cohort study using secondary neuroimaging, genetic, and behavioral data.
Research sample	This longitudinal cohort study used data from the Adolescent Brain Cognitive Development (ABCD) Study, a nationwide study of 11,868 youth aged 9–10 years at baseline recruited from 21 U.S. sites. At baseline, the mean age was 9.91 years and 11.95 years at follow-up (Year 2); 47.5% of participants at baseline and 45.9% at Year 2 were female. The mean BMI increased from 18.73 (SD = 3.96) to 20.49 (SD = 4.45). Participants self-identified as White (52.8%), Black (14.9%), Hispanic (19.8%), Asian (1.9%), or Multiracial (10.6%). Parental education and household income distributions reflected broad socioeconomic diversity. The cohort is broadly representative of U.S. children aged 9–10 years, and data were obtained from the National Institute of Mental Health Data Archive (NDA; ABCD Release 5.1).

Sampling strategy	Participants were recruited through public and private elementary schools using a probability sampling approach stratified by age, sex, race/ethnicity, socioeconomic status, and urbanicity, with oversampling of underrepresented groups to enhance representativeness of the U.S. population. Of the 11,868 participants in the original ABCD cohort, 140 with missing baseline MRI data, 476 with missing PGS_CRP data, 36 from a site that withdrew, and 2 with missing genetic ancestry data were excluded, yielding a final analytic sample of 11,214. Participants were stratified by genetic ancestry (European [n = 6,336] vs. non-European [n = 4,878]) based on genetic ancestry score thresholds (≥0.8 vs. <0.8) to mitigate population stratification bias. No formal sample-size calculation was performed; the ABCD cohort's large, nationally representative design and multi-site recruitment were determined a priori to ensure adequate statistical power to detect small-to-medium effects in developmental and neurobiological outcomes. Within each ancestry group, the majority of participants (European n = 4,588; non-European n = 3,158) completed both baseline and Year 2 assessments. Meta-analytic methods were used to integrate findings across ancestry groups, enhancing generalizability of results.
Data collection	This study used secondary data from the Adolescent Brain Cognitive Development (ABCD) Study, which collected data at 21 U.S. sites using standardized protocols. Trained research staff administered parent- and youth-report questionnaires and acquired 3T MRI scans (Siemens, GE, and Philips systems) following harmonized procedures. All data underwent centralized quality control by the ABCD Data Analysis and Informatics Resource Center and were obtained in de-identified form from the National Institute of Mental Health Data Archive (NDA; ABCD Release 5.1). The present investigators did not participate in data collection.
Timing	09/01/2016 - 02/15/2021
Data exclusions	The ABCD Study excluded participants for non-fluency in English, absence of a guardian fluent in English or Spanish, major medical or neurological conditions, gestational age <28 weeks or birthweight <1,200 grams, MRI contraindications, history of traumatic brain injury, current schizophrenia, moderate to severe autism spectrum disorder, intellectual disability, or alcohol/substance use disorder.<br><br>For the current analysis, the baseline cohort excluded 140 participants missing MRI data, 476 missing PGS_CRP data, 36 screened at a study site that withdrew, and 2 missing genetic ancestry data, yielding 11,214 participants. The two-year follow-up cohort excluded 2,881 with missing MRI data, 267 missing PGS_CRP data, and 2 missing genetic ancestry data.
Non-participation	Of 11,214 participants at baseline, 7,823 completed the two-year follow-up, reflecting a 70% retention (30% attrition) rate.
Randomization	Participants were not randomized; this was an observational study. Analyses controlled for key covariates (age, sex, BMI, socioeconomic factors, and ancestry components) and stratified by genetic ancestry to reduce bias.

Reporting for specific materials, systems and methods

We require information from authors about some types of materials, experimental systems and methods used in many studies. Here, indicate whether each material, system or method listed is relevant to your study. If you are not sure if a list item applies to your research, read the appropriate section before selecting a response.

Materials & experimental systems

n/a	Involved in the study	
☒	☐	Antibodies
☒	☐	Eukaryotic cell lines
☒	☐	Palaeontology and archaeology
☒	☐	Animals and other organisms
☒	☐	Clinical data
☒	☐	Dual use research of concern
☒	☐	Plants

Methods

n/a	Involved in the study	
☒	☐	ChIP-seq
☒	☐	Flow cytometry
☐	☒	MRI-based neuroimaging

Plants

Seed stocks	NA
Novel plant genotypes	NA
Authentication	NA

Magnetic resonance imaging

Experimental design

Design type	The ABCD MRI protocol included both resting-state and task-based fMRI. The task-based scans used event-related designs for the Monetary Incentive Delay (MID) and Stop Signal Task (SST), and a block design for the emotional n-back (EN-back) working memory task.designs for the Monetary Incentive Delay (MID) and Stop Signal Task (SST), and a block design for the emotional n-back (EN-back) working memory task. Details are not repeated in the main text to avoid redundancy, but are fully described in the ABCD imaging acquisition papers cited in the manuscript (Refs. 44 and 56).
Design specifications	Each session included two resting-state fMRI runs (~5 min each) and three task-based paradigms (MID, SST, EN-back). MID: event-related; ~50 trials per run (2 runs); 4–6 s trial duration; jittered 1.5–4 s inter-trial interval. SST: event-related; 180 trials per run (2 runs), including ~30 stop trials (16.7%); 1 s trial duration; 1.7–3 s inter-trial interval. EN-back: block design; 8 blocks per run (2 runs) alternating 0-back and 2-back; ~30 s per block with 15–20 s fixation between blocks. Details are not repeated in the main text to avoid redundancy, but are fully described in the ABCD imaging acquisition papers cited in the manuscript (Refs. 44 and 56).
Behavioral performance measures	Behavioral data were collected using computerized response boxes during all task-based fMRI scans.<br>MID: accuracy (hit rate) and reaction time.<br>SST: stop-signal reaction time (SSRT), accuracy on "Go" and "Stop" trials.<br>EN-back: accuracy and reaction time on 0-back and 2-back blocks.<br>Mean accuracy and reaction times across participants confirmed expected task engagement and performance consistency. Details are not repeated in the main text to avoid redundancy, but are fully described in the ABCD imaging acquisition papers cited in the manuscript (Refs. 44 and 56).

Acquisition

Imaging type(s)	Structural MRI (T1-weighted), functional MRI (resting-state and task-based fMRI), and diffusion MRI.
Field strength	3 Tesla
Sequence & imaging parameters	Detailed MRI acquisition parameters (e.g., TR, TE, TI, voxel size, sequence type, and field strength) are specified in the ABCD Study protocol, which our analyses followed exactly (references 44 and 56 in the manuscript). Therefore, acquisition details are not repeated in the main text but are available in the cited ABCD documentation
Area of acquisition	Whole brain coverage, including cerebrum and cerebellum.

Diffusion MRI ☒ Used ☐ Not used

Parameters: Multi-shell diffusion MRI was acquired using a single-shot, spin-echo EPI sequence with 96 diffusion directions across four b-values (500, 1000, 2000, and 3000 s/mm²), plus 7 interspersed b0 volumes. The sequence used no cardiac gating. (standardized ABCD Study diffusion protocol, Refs 44 and 56)

Preprocessing

Preprocessing software	ABCD pipeline implemented in FreeSurfer v7.1.1, FSL, and AFNI, managed by the ABCD Data Analysis and Informatics Resource Center (DAIRC).
Normalization	Images were aligned to the participant's native space and then normalized to MNI152 standard space.
Normalization template	Montreal Neurological Institute (MNI152) atlas.
Noise and artifact removal	Gradient nonlinearity distortion correction, B0 inhomogeneity correction, motion correction, and intensity normalization performed as part of the ABCD preprocessing pipeline. fMRI data underwent ICA-based artifact removal (ICA-FIX) and nuisance regression to reduce motion and physiological noise.
Volume censoring	Volumes with framewise displacement >0.9 mm were censored from fMRI analyses; participants with >50% of frames censored were excluded, per ABCD preprocessing standards.

Statistical modeling & inference

Model type and settings	Linear mixed-effects models tested associations between PGS_CRP, age, and early-life infection, including random intercepts for individual, family, and site. Continuous predictors were standardized.
Effect(s) tested	Main and interaction effects of PGS_CRP × age × early-life infection on cortical thickness and psychopathology. Structural equation models (SEM) tested cortical thinning as a mediator of PGS_CRP effects.

Specify type of analysis: ☐ Whole brain ☐ ROI-based ☒ Both

Anatomical location(s)

Anatomical regions were defined using the Desikan–Killiany cortical atlas, applied through automated surface-based labeling in FreeSurfer v7.1.1 to derive 34 cortical regions per hemisphere and global mean cortical thickness measures.

Statistic type for inference

(See Eklund et al. 2016)

Voxel-wise statistical inference was performed within each ROI, with results corrected for multiple comparisons using False Discovery Rate (FDR; $p < 0.05$, two-tailed) across all tested regions. No cluster-wise correction was applied.

Correction

Multiple comparisons were controlled using the False Discovery Rate (FDR) at $p < 0.05$ (two-tailed) across all cortical regions.

Models & analysis

n/a	Involved in the study
☒	☐ Functional and/or effective connectivity
☒	☐ Graph analysis
☐	☒ Multivariate modeling or predictive analysis

Multivariate modeling and predictive analysis

Independent variable: PGS_CRP (polygenic score for C-reactive protein).
Feature extraction: Global mean cortical thickness change (T2–T0) derived from FreeSurfer parcellations.
Dimension reduction: Not applicable (single global mediator variable).
Model: Structural equation modeling (SEM) tested indirect effects of PGS_CRP on psychopathology via cortical thickness change.
Evaluation metrics: Standardized path coefficients, bootstrapped confidence intervals (1,000 samples), and model fit indices (CFI, TLI, SRMR).
